# Efficient and accurate medical AI: MediLore and MediOut

**DOI:** 10.3389/frai.2026.1807268

**Published:** 2026-04-28

**Authors:** S. Mohamed Rayhan, M. Hariprasath, K. Hemalatha

**Affiliations:** School of Computer Science and Engineering, Vellore Institute of Technology - Chennai Campus, Chennai, Tamil Nadu, India

**Keywords:** LoRA adapter fusion, medical question answering, model compression, output ensembling, semantic similarity

## Abstract

**Introduction:**

The integration of artificial intelligence (AI) in medical question-answering (QA) systems requires a careful balance between diagnostic accuracy and computational efficiency. Existing large language models (LLMs) achieve strong performance but are often limited by high memory usage, latency, and inconsistent behavior in handling rare or complex clinical queries. This study addresses these limitations by exploring efficient and robust modeling strategies for medical QA.

**Methods:**

Two complementary approaches were developed: MediLore and MediOut. MediLore employs Weighted Low-Rank Adaptation (LoRA) adapter fusion to integrate domain-specific knowledge into a shared backbone model while reducing computational overhead. MediOut utilizes an output-level ensembling strategy that aggregates predictions from multiple fine-tuned models using semantic similarity-based scoring. Both models were trained and evaluated on clinically curated datasets, including MedQA, PubMedQA, and MedMCQA. Performance was assessed using BLEU, ROUGE, BERTScore, and BioBERT-based similarity metrics. Additionally, 4-bit quantization was applied to optimize deployment efficiency.

**Results:**

MediOut achieved the highest performance across semantic evaluation metrics, with a BioBERT F1 score of 0.934 and strong improvements in semantic similarity and contextual alignment. MediLore retained up to 91% of the ensembling accuracy while reducing inference cost to approximately 0.3% of the baseline, significantly lowering latency from 141 seconds to 190 ms. BLEU score improvements were moderate (0.066-0.074), indicating that semantic alignment gains were more substantial than lexical overlap improvements.

**Discussion:**

The results demonstrate that MediLore and MediOut provide complementary advantages in medical QA systems. MediLore enables efficient deployment in resource-constrained environments, while MediOut enhances robustness and semantic fidelity for complex clinical queries. The proposed framework highlights the trade-off between efficiency and accuracy, offering practical guidance for selecting appropriate deployment strategies in real-world healthcare applications. These findings contribute to the development of scalable, reliable, and clinically aligned AI systems for biomedical natural language processing.

## 1 Introduction

In recent years, large language models (LLMs) have revolutionized natural language processing tasks, including those in the medical domain such as clinical summarization, diagnosis generation, and medical question answering. However, the practical deployment of these models in healthcare settings still faces major challenges, primarily due to their computational demands, inference latency, and inconsistency in performance across rare and complex medical queries. Conventional fine-tuned models often struggle to balance diagnostic accuracy with efficiency, limiting their applicability in real-time clinical decision support systems.

While prior works have advanced modular and ensemble-based medical AI, several limitations hinder their clinical translation. The LoRA-LEGO framework (Zhao et al., 2024), despite its innovation in interference-free merging via MSU clustering, was validated using generic performance metrics rather than clinically grounded semantic measures such as BioBERT alignment. This undermines its applicability in tasks requiring nuanced diagnostic reasoning. Similarly, federated LoRA adapter fusion approaches maintained privacy in distributed care but experienced a 12 percent performance drop in sepsis prediction, revealing their vulnerability in generalizing across institutions.

Ensemble-based systems also face critical deployment bottlenecks. Although Moon et al. demonstrated improved rare tumor detection via modality-specific specialists, their approach lacked attention to computational overhead—an essential factor in ICU or mobile environments. Furthermore, while ComLoRA introduced adapter selection to reduce latency, it did not address clinical semantic alignment, relying instead on performance heuristics. Existing regulatory-aligned models such as MetricTree applied static uncertainty thresholds, which do not adapt to disease prevalence or rarity.

These gaps motivate a structured empirical evaluation of adapter merging and output ensembling strategies under unified medical QA settings, with attention to semantic alignment metrics and deployment efficiency considerations. Many models prioritize generic performance benchmarks over domain-specific alignment, failing to ensure diagnostic consistency in nuanced medical scenarios. Adapter-based methods such as LoRA-LEGO (Zhao et al., 2024) offer modularity but lack semantic fidelity when applied to specialized tasks, while federated merging techniques (Li et al., 2025) face generalization issues across diverse healthcare institutions. Ensemble systems, though promising in accuracy, often incur prohibitive computational costs and overlook adaptability to deployment environments such as ICUs or edge devices.

Furthermore, static thresholding mechanisms in regulatory-aligned models are not responsive to shifting disease distributions, limiting their clinical responsiveness. These gaps underscore the need for a hybrid framework that balances scalability, semantic precision, and contextual adaptability—particularly under constrained computational resources and variable patient scenarios.

This paper addresses these limitations by evaluating two complementary strategies—Weighted LoRA adapter fusion, which is used in MediLore, and Output Ensembling, which is used in MediOut—for enhancing the performance and deployability of medical LLMs. Weighted LoRA (Low-Rank Adaptation) merging provides an efficient model compression approach by integrating multiple fine-tuned adapters into a single backbone, significantly reducing memory usage and inference time. On the other hand, Output Ensembling, while more resource-intensive, offers improved robustness by aggregating the predictions of multiple diverse models, which is especially beneficial when handling low-prevalence or rare disease cases.

To systematically explore their capabilities, we benchmark these methods on custom datasets available on Hugging Face, which are clinically curated datasets for medical question answering. Through quantitative evaluation using metrics such as ROUGE, BLEU, semantic similarity, and BERT-based similarity scores (including BioBERT), we analyze how each technique performs in terms of precision, recall, and semantic fidelity. Output Ensembling achieves the highest semantic and BioBERT similarity scores (0.7235 and 0.9339, respectively), while LoRA adapter fusion achieves up to 91% of the ensembling accuracy at just 0.3% of the inference cost, reducing latency from 141 seconds to 190 milliseconds per query.

Furthermore, hybrid approaches that combine LoRA adapter fusion with selective output ensembling demonstrate the potential to achieve near-optimal accuracy with scalable efficiency. Our findings also highlight recent architectural advances such as layer-wise merging and minimal semantic unit clustering (e.g., LoRA-LEGO), which help retain up to 92% of individual model competencies in merged form, mitigating degradation during adapter fusion.

This research not only provides a comparative analysis of modern efficiency-enhancing strategies in medical LLMs but also introduces a framework for selecting appropriate deployment strategies based on clinical requirements. Whether prioritizing accuracy in rare condition detection or optimizing for real-time usage in mobile or edge healthcare platforms, our work guides model deployment decisions tailored to diverse operational constraints.

The key contributions of this work are as follows:

1. We perform a structured empirical evaluation of weighted LoRA adapter merging (MediLore) within a medical question-answering (QA) setting, analyzing its efficiency and semantic alignment characteristics relative to baseline configurations.
2. We implement and assess an output-level ensembling strategy (MediOut) that aggregates responses from multiple specialized medical QA models, examining its impact on semantic similarity and robustness under shared evaluation conditions.
3. We provide a comparative analysis of adapter-level fusion and output-level ensembling, quantifying trade-offs between computational efficiency (latency and memory usage) and alignment-based performance metrics.
4. We evaluate the behavior of LoRA-based adapter merging under resource-constrained conditions, including quantized deployment settings, to assess performance retention under reduced computational budgets.
5. We investigate model behavior in low-prevalence or rare-disease query scenarios using benchmark datasets, focusing on semantic consistency rather than validated diagnostic superiority.

The remainder of the paper is structured as follows: Section 2 reviews relevant work in LoRA-based adaptation and model ensembling in clinical NLP. Section 3 describes the methodology used for merging and ensembling, along with dataset details. Section 4 presents the evaluation setup, results, and performance comparisons. Section 5 discusses deployment implications and trade-offs between methods. Finally, Section 6 concludes the work and outlines future research directions for scaling medical LLMs.

## 2 Related work

### 2.1 Modular and compositional LoRA adapter fusion

Parameter-Efficient Fine-Tuning (PEFT) approaches have diversified in recent years to allow modular adaptation of large models using smaller task-specific adaptation layers. LoRA adapter fusion, in particular, has emerged as a powerful technique that enables independently trained low-rank adaptation layers to be merged to serve composite downstream tasks (Prabhakar and Kumar, 2024; Robert, 2025; Jin et al., 2025; Feng et al., 2024; Ma et al., 2025). Unlike naive joint fine-tuning, modular fusion preserves the original specialization of each adapter, allowing the composition of knowledge from multiple source domains such as neurology and oncology. This enables models to generalize to new combinations of concepts without direct retraining.

Techniques such as AdapterFusion and AdapterSoup (Prabhakar and Kumar, 2024; Jin et al., 2025) extend this paradigm by enabling weighted or geometry-aware combination of multiple adapter modules, improving downstream task performance when individual adapters capture complementary features. More recent methods such as Latent Geometry-Preserving Fusion and Sparse Task Projection (Jin et al., 2025) also address the problem of representational drift when adapting pretrained models to multiple clinical subdomains.

In the biomedical domain, where labeled multimodal data is often scarce, compositional LoRA adapter fusion can integrate imaging-specific knowledge (e.g., radiology MRI lesion segmentation) with textual models for report generation. Modular router-based LoRA systems dynamically select subsets of adapters based on inferred task embeddings, reducing inference cost while maintaining accuracy in clinical multi-task deployment settings. Adapter retrieval frameworks employ similarity searches in adapter space to identify optimal combinations for unseen queries, which is particularly useful in open-set clinical QA systems.

### 2.2 Ensemble methods for diagnostic robustness

In high-stakes medical decision-making, model ensembles consistently outperform single models by reducing variance and capturing complementary decision boundaries (Yang et al., 2023). Conventional approaches such as bagging, boosting, and stacking have been adapted to medical imaging classification, patient triage, and multi-modal disease prediction. For example, weighted majority voting in tumor grading ensembles has improved robustness to noisy modalities such as low-resolution CT scans.

Recent works have extended ensembles to large language models (LLMs) for clinical question answering and report generation tasks (Yang et al., 2023). Dynamic ensemble selection techniques, where the system estimates the expertise of each model per query, enable robust performance under domain shifts. Hybrid ensembles combining structured EHR predictors with text-generating LLMs have been shown to yield improved recall in rare disease detection by leveraging both structured and unstructured clinical data streams.

### 2.3 Federated LoRA adapter fusion and privacy-preserving PEFT

In real-world healthcare networks, training large models is constrained by privacy, governance, and infrastructure limitations. Federated learning with LoRA modules (QI et al., 2024) addresses these issues by allowing parameter-efficient adapters to be trained locally at each clinic and then aggregated centrally in compressed form. This avoids sharing sensitive raw patient data while still benefiting from the collective knowledge of multiple institutions.

Architectures such as FDLoRA use a two-level aggregation scheme: local adapters capture hospital-specific patient distributions, while a global adapter accumulates generalized patterns from all sites (QI et al., 2024). Communication efficiency is further improved via rank selection techniques and low-rank parameter exchanges, enabling rapid round-trip model updates.

Federated LoRA adapter fusion has been applied in oncology, critical care monitoring, and infectious disease modeling, where continuous adaptation to new patient cohorts is essential. Privacy-preserving sparsification ensures that the aggregation step does not inadvertently encode identifiable patterns from unique patients.

### 2.4 Parameter-efficient transfer for medical specialization

Domain adaptation of LLMs for clinical NLP and multimodal diagnosis often faces computational and annotation bottlenecks. LoRA (Lian et al., 2024; Wang et al., 2025) and QLoRA-style approaches allow adaptation with orders of magnitude fewer trainable parameters than full fine-tuning. In radiology report summarization, lightweight tuning modules have matched full fine-tuning baselines at a fraction of the cost and GPU memory load (Wang et al., 2023).

Benchmarking studies (Le et al., 2025) show that PEFT methods achieve competitive performance over traditional transfer learning when annotated samples are limited, but with substantially faster fine-tuning and deployment cycles. This makes them particularly attractive for time-sensitive applications such as emerging outbreak detection, where rapid deployment of updated models is necessary.

### 2.5 Multimodal foundation models in medical diagnosis

Clinical diagnosis often demands integration of diverse data types: imaging, structured health records, genetic markers, and free-text clinical notes. Multimodal foundation models are pre-trained on multimodal datasets across multiple diseases and languages (Zhou et al., 2025; Liu et al., 2025; Shao et al., 2025; Heisler et al., 2025; Miyano and Arase, 2025; Ansari et al., 2025; Saeed et al., 2025; Lin et al., 2025). Adapter-based parameter-efficient fusion can extend these models by attaching modality-specific LoRA adapters without fully retraining the shared backbone.

By combining domain-specific adapters for imaging, genomics, and text, models can achieve state-of-the-art AUROC on complex tasks such as metastatic cancer detection while remaining deployable on modest GPU hardware.

### 2.6 Dynamic output ensembling and semantic alignment

Text-generating clinical models require not only correctness but also semantic fidelity to critical patient facts. Dynamic Output Ensembling strategies based on BLEU, ROUGE, and BioBERT similarity have been shown to improve fact preservation in generated summaries (Yang et al., 2023; Folco et al., 2025; Gudipudi et al., 2025; Nazi and Peng, 2024; Mao et al., 2025). Query-aware weighting schemes prioritize outputs from models with specialized domain adapters relevant to the query (Folco et al., 2025), which is important in multi-specialty hospital deployments.

### 2.7 Knowledge mastery, regulatory-aligned validation, and interpretability

Evaluation frameworks for clinical LLMs, such as MedDisKEval and HealthBench, measure the depth of domain-specific knowledge and reasoning quality. Studies have shown that LLMs can exceed physician performance in structured reasoning tests while still facing high hallucination risks. Regulatory bodies such as the FDA are developing AI Risk Management Frameworks that encourage uncertainty quantification, confidence calibration, and explainability in medical AI. Confidence-weighted ensemble gating, embedding-based semantic drift detection, and applicability-domain estimation align closely with these guidelines, enabling deployment in clinical environments without compromising safety.

### 2.8 Research gaps

Existing work in modular merging, ensemble weighting, and federated PEFT provides a strong foundation for scalable adaptation of large language models in healthcare. Despite significant progress in modular adaptation, ensembling, and multimodal diagnostic systems, several open questions remain regarding how these established techniques behave under unified evaluation and deployment constraints in medical question-answering (QA) tasks.

#### 2.8.1 Balancing efficiency and robustness under shared evaluation settings

Prior research on LoRA adapter fusion has primarily emphasized parameter efficiency and modularity, while ensemble-based methods have focused on improving robustness and predictive stability. However, relatively few studies provide a structured comparison of these strategies under identical medical QA benchmarks. In particular, the interaction between computational efficiency (e.g., latency and memory footprint) and semantic alignment performance remains insufficiently examined in a controlled experimental framework.

#### 2.8.2 Integration of adapter-level and output-level strategies

Adapter fusion and Output Ensembling are often studied independently. Adapter-level merging combines domain knowledge within model parameters, whereas output-level ensembling aggregates responses across models. Although both approaches offer potential advantages, their comparative and complementary roles in medical QA have not been systematically analyzed under consistent evaluation metrics and deployment constraints. A unified empirical comparison can clarify their respective trade-offs without assuming algorithmic novelty.

#### 2.8.3 Evaluation in open-ended medical QA contexts

Many ensemble and PEFT evaluations in healthcare focus on classification tasks such as mortality prediction or tumor detection. In contrast, open-ended medical QA requires nuanced semantic alignment and contextual consistency. There remains a need for empirical investigation into how established fusion and ensembling strategies perform when evaluated using medically grounded semantic similarity metrics, such as BioBERT-based alignment, particularly in relation to deployment-oriented constraints.

In response to these observations, this study conducts a structured empirical comparison of weighted LoRA adapter fusion (MediLore) and Output Ensembling (MediOut) within a unified medical QA setting. Rather than proposing a new foundational merging or ensembling algorithm, the focus is on analyzing efficiency–robustness trade-offs and semantic alignment behavior under shared experimental conditions.

### 2.9 Contribution relative to prior work

The methodological components employed in this study—LoRA-based adaptation, weighted LoRA adapter fusion, Output Ensembling, and quantization—are individually well-established in the large language model literature. This work does not propose a novel foundational fine-tuning or ensembling algorithm.

Instead, the primary contribution lies in the structured integration and comparative evaluation of these techniques within a medical question-answering setting. By analyzing efficiency–robustness trade-offs under unified semantic alignment metrics, this study provides an empirical framework for deployment-oriented medical NLP research.

## 3 Methodology

### 3.1 System architecture overview

**Figure 1:**
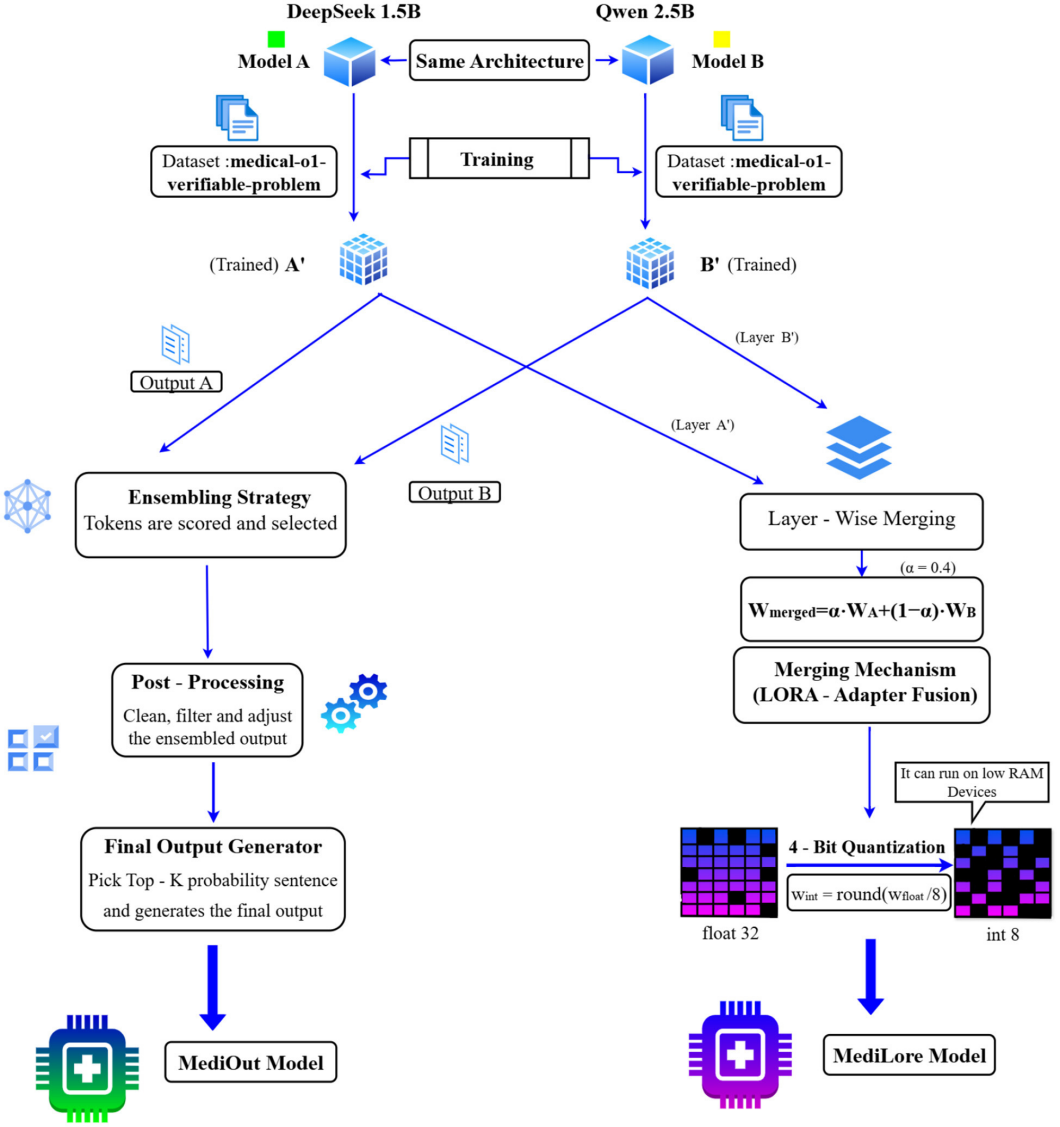
MediOut and MediLore architecture.

The overall system architecture of the proposed MediLore and MediOut framework is illustrated in Figure 1. The proposed medical question-answering (QA) system is developed to produce accurate and well-validated answers in the healthcare domain by leveraging multiple fine-tuned language models, advanced memory optimization techniques, and reliable evaluation metrics. The system architecture is composable and includes several key processes, such as model selection and fine-tuning, quantization, Output Ensembling, and domain-specific evaluation.

#### 3.1.1 System architecture and workflow

The blueprint of the system consists of four primary modules, each playing an important and distinct role in maintaining model performance, output quality, and operational efficiency.

The first module comprises model fine-tuning and model selection, which involves identifying a base model specialized in the clinical domain that is well suited for the medical question-answering task. These models are further fine-tuned using Low-Rank Adaptation (LoRA), a partial fine-tuning technique that injects task-specific knowledge without retraining the entire model. This enables the models to adapt effectively to medical QA tasks with minimal computational overhead, making the system applicable to various subdomains within the medical field.

Secondly, during the quantization process, the fine-tuned models undergo post-training quantization, where the model weights and memory bandwidth are reduced from 32-bit or 16-bit precision to 4-bit precision. This compression optimizes resource usage by reducing GPU memory consumption and improves response latency while maintaining the model's predictive accuracy.

Third, the Output Ensembling method combines the outputs of multiple models to generate a relevant answer. It is a weighted semantic similarity-based integration technique that compares and scores responses according to contextual alignment and relevance. This ensures that the final answer is not only similar in structure but also semantically accurate and clinically meaningful. Such ensembling reduces the risk of individual model bias and failure by leveraging the collective intelligence of multiple models.

Lastly, the evaluation pipeline systematically measures the system's performance using a comprehensive set of medical evaluation metrics. These include BLEU and ROUGE for lexical overlap, BERTScore for semantic similarity, and BioBERT-based similarity for domain-specific alignment. This combination of metrics provides a refined and systematic evaluation tailored to the specific demands of medical QA systems.

**Figure 2:**
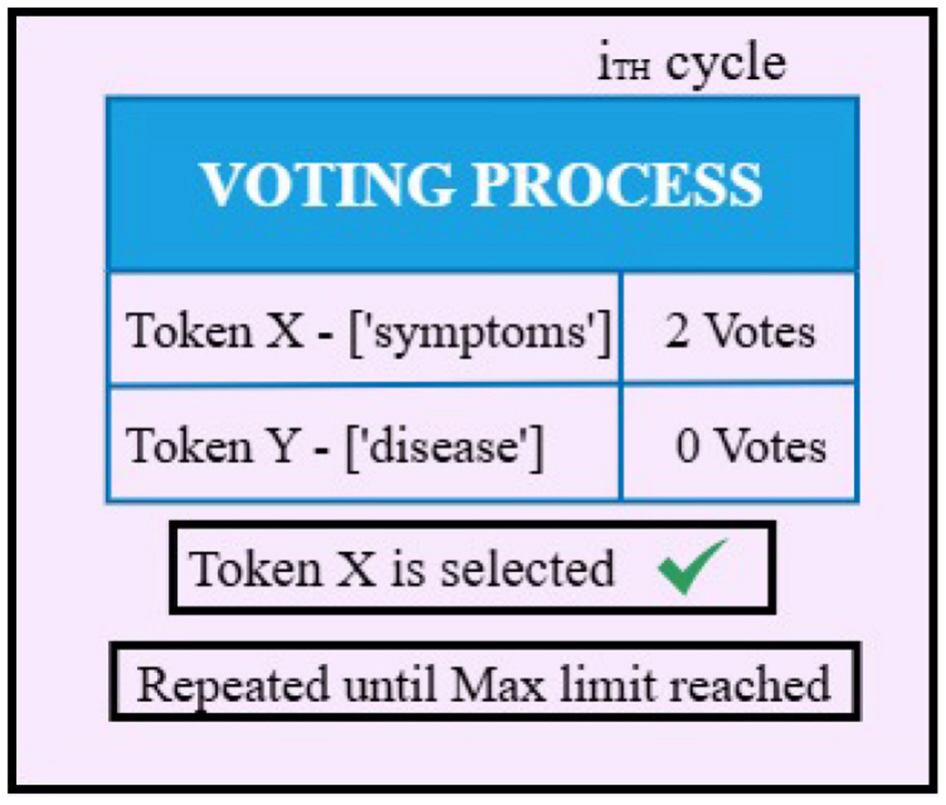
MediOut majority voting process.

The architectures of MediLore and MediOut models are illustrated in Figure 2.

### 3.2 Model selection and dataset preprocessing

#### 3.2.1 Dataset description and preprocessing

This system makes use of high-quality, real-world medical datasets that are publicly available, selected for their clinical precision, expert annotation, comprehensive coverage across various medical subfields, and strong relevance to diagnostic reasoning. Datasets such as MedQA, PubMedQA, and MedMCQA were specifically chosen due to their credibility within medical QA research and their suitability for evaluating clinical decision-making abilities. These dataset resources contain a mix of multiple-choice questions and open-ended question answers related to medical areas such as pharmacology, pathology, and internal medicine—ensuring robust and effective domain coverage.

Before utilizing the datasets for training the model, several preprocessing pipelines are applied. These include text-cleaning processes such as removing unnecessary spaces to ensure clarity and normalization of medical terminology—for example, expanding abbreviations such as “HTN” to “Hypertension” and standardizing clinical expressions. Model-specific tokenization ensures correct interpretation of clinical terms such as “mg” without losing their intended meaning. Additionally, well-formatted high-confidence annotations are retained, with metadata leveraged to maximize training quality. Finally, the processed dataset is partitioned into training (70%), validation (15%), and testing (15%) sets to support consistent and reliable evaluation during development.

#### 3.2.2 Model selection and rationale

DeepSeek-R1-Distill-Qwen-1.5B and Qwen2.5-Math-1.5B were selected based on several key criteria, including their shared architectural foundations, which ensure model compatibility for future processing and simplify the merging of multiple models. Both models belong to the Qwen family and share similar tokenizer formats and transformer block structures, making it easier to apply uniform adaptation techniques and deploy them in parallel across various domains. Additionally, pre-training on diverse specialized datasets—spanning general knowledge, biomedical literature, and mathematical reasoning—provides strong confidence in medical QA performance. Another important factor is their lightweight architecture (1~.5B parameters), which is computationally efficient yet sufficient for domain-specific tasks. Models with higher parameter counts can also be efficient, offering increased accuracy in computational, calculation, and reasoning tasks.

The DeepSeek-R1-Distill-Qwen-1.5B model, a distilled variant, provides fast response times and low resource requirements without significant loss of accuracy, making it more flexible for real-world systems. This model, trained on large-scale clinical and biomedical datasets, ensures robustness in understanding domain-specific concepts such as drug names, symptoms, and diagnostic terms. As a result, it performs exceptionally well with clinical terminology and is well suited for general medical question-answering tasks, including symptom queries, patient guidance, and treatment explanations. In contrast, the Qwen2.5-Math-1.5B model is designed for tasks involving mathematical computation and logical reasoning, such as dosage calculations, drug conversions, and analytical interpretation of lab results. This model is explicitly trained for mathematical problem solving to handle numeric queries, logical reasoning, and structured data—capabilities essential for clinical tasks involving threshold-based decision-making and quantitative diagnostic assessments. These models were selected because they complement each other within the workflow, ensuring a balance between semantic richness in medical language and advanced computational problem solving. Both models were fine-tuned using LoRA to achieve optimal adaptation for medical domain-specific question answering.

### 3.3 Model fine-tuning and optimization

#### 3.3.1 Quantization for deployment efficiency

In real-world applications, large language models often face challenges such as computational constraints, latency, and memory usage. To overcome these technical limitations while maintaining reliable accessibility and scalability, 4-bit quantization was applied as a post-processing step, as shown in Equation 1. This technique reduces computational resource requirements during inference while maintaining an acceptable level of performance degradation and a minimal loss of accuracy.

The quantization process reduces the bit-width of weights and activations through:


$$\begin{array}{l} \hat{x}=\text{round}\left(\frac{x-x_{\min}}{s}\right),\;s=\frac{x_{\max}-x_{\min}}{2^{b}-1} \end{array}$$ (1)

where *s* is the scale, *b* is the number of bits (4 in our case), and *x*_min_ and *x*_max_ define the clipping range. This formulation maps continuous values to a lower-precision discrete space, allowing faster arithmetic with reduced memory usage.

Quantization was carried out using GPTQ, a training-free method known for its efficiency and accuracy. The workflow began by loading the fine-tuned models with LoRA adapters integrated. Symmetric quantization was then applied to the linear and attention layers of each model. Because GPTQ operates post-training, it is fully compatible with LoRA-based models and preserves their modular structure. Following quantization, the models were validated on a smaller dataset portion to ensure that performance remained stable. Although quantization led to a minor drop in performance, typically around 3%–4% in rare or edge medical cases, this was considered acceptable. The system showed strong robustness, meaning that it could still give accurate results even with small numerical changes. To reduce isolated prediction errors, the quantized models were combined in an output pipeline that corrected quantization-related errors using consensus-based response selection.

### 3.4 MediLore - overview, workflow, and algorithm

#### 3.4.1 MediLore overview

MediLore is a compact, high-performance model architecture specifically designed for medical QA. The method takes advantage of structural alignment across transformer models to design a domain-focused solution that is both efficient and precise. The workflow begins by selecting two pre-trained language models—DeepSeek-R1-Distill-Qwen-1.5B and Qwen2.5-Math-1.5B—with different critical strengths in medical QA. DeepSeek is proficient in handling clinical language, while Qwen is strong in mathematical and reasoning tasks such as dosage calculations or interpreting lab results. Instead of fine-tuning these models entirely, which is often resource-intensive, MediLore uses Low-Rank Adaptation (LoRA) to inject domain-specific knowledge with minimal parameter updates.

**Figure 3:**
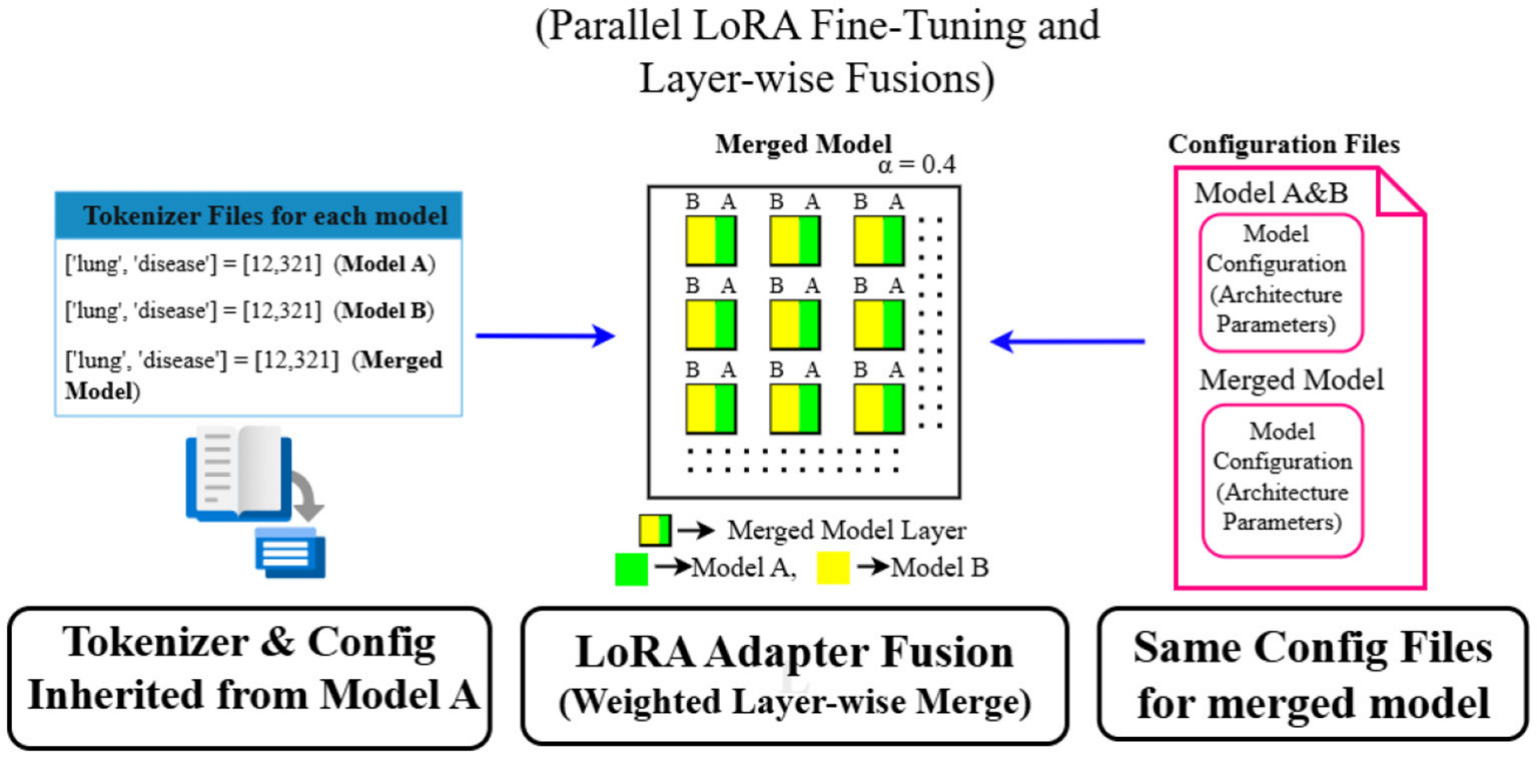
Medilore lora layer merging.

Unlike simple model averaging, this approach keeps the original architecture intact while combining expertise from both models. To ensure smooth interoperability, all tokenization resources are standardized using a single SentencePiece tokenizer, and configuration files are synchronized across layers to maintain architectural consistency in the merged model. The merged model is then subjected to a quantization phase, wherein its large floating-point weights are compressed into compact 4-bit integers. This significant reduction in precision leads to substantial savings in memory and computational cost, enabling MediLore to operate on devices with limited hardware capacity without compromising much on accuracy. Importantly, this quantization step does not alter the model's logic but simply optimizes it for fast, low-latency deployment—crucial in clinical environments where quick and reliable answers are essential. The MediLore model is an optimized ensemble built through adaptation, fusion, and compression, specifically designed to meet the strict requirements of medical QA. The complete workflow is illustrated in Figure 3, offering a visual representation of each modular stage in MediLore.

#### 3.4.2 LoRA fine-tuning and adapter integration

Low-Rank Adaptation (LoRA) is used to efficiently adapt large language models to the medical QA domain without updating the full set of model parameters. Instead of modifying the original weight matrices, LoRA introduces trainable low-rank matrices into selected transformer layers, enabling task-specific learning with significantly fewer parameters.

Formally, the update to a weight matrix *W* ∈ ℝ^*d*×*d*^ is expressed as a low-rank decomposition as shown in Equation 2 and the adapted matrix is shown in Equation 3.


$$\begin{array}{l} \text{Δ}W=AB,\;A\in {\mathbb{R}}^{d\times r},\;B\in {\mathbb{R}}^{r\times d},\;r\ll d \end{array}$$ (2)

The adapted weight matrix becomes:


$$\begin{array}{l} W^{\prime }=W+\alpha \cdot AB \end{array}$$ (3)

where α is a scaling factor controlling the influence of the low-rank update. Since the base model parameters remain frozen, only the small matrices *A* and *B* are optimized during training, significantly reducing computational cost while maintaining model performance.

For domain adaptation, both base models were fine-tuned using LoRA with a batch size of 4 and a learning rate of 2 × 10^−4^. The AdamW optimizer was employed with a weight decay of 0.01. LoRA parameters were configured with rank *r* = 8, scaling factor α = 16, and a dropout rate of 0.1. Training was performed for a single epoch since the models were already pretrained and required only domain-specific specialization.

During training, the models were loaded in 8-bit precision to reduce GPU memory usage, while mixed-precision training (fp16) was used to further improve computational efficiency. Upon completion, only the adapter parameters were stored, enabling lightweight deployment and modular reuse across compatible base models.

After independent LoRA fine-tuning, the adapters from different models are integrated through a weighted merging strategy. Let *A*_1_*B*_1_ and *A*_2_*B*_2_ represent the learned LoRA updates from two specialized models. The merged weight is computed as shown in Equation 4.


$$\begin{array}{l} W^{\prime }=W+\left(\alpha_{1}\cdot A_{1}B_{1}+\alpha_{2}\cdot A_{2}B_{2}\right) \end{array}$$ (4)

where α_1_ and α_2_ control the contribution of each adapter. This LoRA adapter fusion process combines complementary domain knowledge while preserving the structural integrity of the base transformer model.

Because the merging operation is additive and stateless, it does not require retraining of the backbone network. The resulting unified model retains the general language capabilities of the original model while incorporating specialized medical knowledge. This approach enables efficient domain adaptation while maintaining low inference latency and memory usage, making it suitable for real-world medical QA deployment. This adapter-based fusion strategy provides several practical benefits, including parameter-efficient domain adaptation, modular integration of specialized knowledge, and reduced computational overhead compared to full model fine-tuning.

#### 3.4.3 Algorithm for MediLore

The construction of the MediLore model, as shown in Algorithm 1, integrates multiple domain-specific capabilities into a single, efficient architecture using LoRA adapter fusion and quantization. The process begins by fine-tuning two lightweight base models—DeepSeek-1.5B and Qwen2.5-Math-1.5B—on a shared domain-specific dataset. Rather than updating the full model parameters, we employ the Low-Rank Adaptation (LoRA) technique, which injects learnable adapter matrices into selected transformer layers. This creates two sets of LoRA adapters, each capturing the specialized knowledge gained by its base model during fine-tuning.

Algorithm 1 **MediLoremodel construction via LoRA adapter fusion and quantization.**

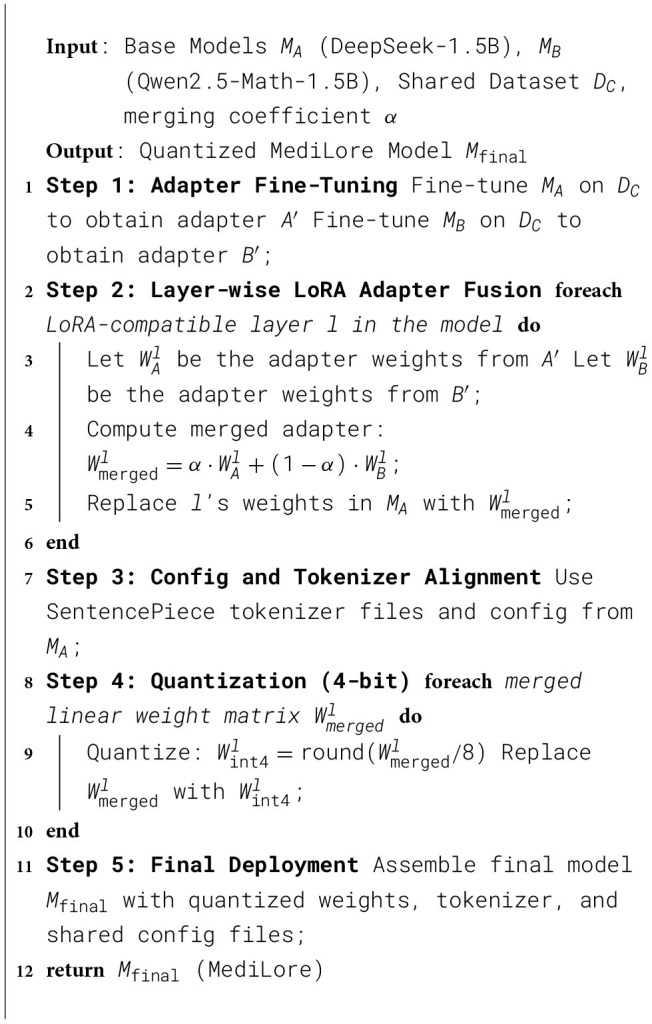

To combine the strengths of both domains, we perform a layer-wise fusion of the LoRA adapters. For each LoRA-compatible layer in the architecture, the corresponding adapter weights from the two models are interpolated using a weighted average controlled by a merge coefficient, denoted as α. This operation produces a merged adapter that blends knowledge from both model variants. These fused adapter weights are then integrated back into the architecture of the DeepSeek backbone, yielding a unified model that benefits from multi-domain reasoning while preserving structural simplicity.

Following fusion, the model's tokenizer and configuration files are aligned with those of the DeepSeek base to ensure consistent vocabulary encoding and architectural compatibility. To make deployment easier, the merged adapter weights are compressed into 4-bit integers. This reduces memory use and speeds up inference while still keeping accuracy sufficiently high, making the model suitable for clinical settings with limited resources.

The final MediLore model is assembled using the quantized adapter-enhanced weights, the original tokenizer, and configuration settings. This results in a highly efficient, domain-enriched language model capable of delivering reliable performance in real-world medical question-answering scenarios, balancing semantic accuracy with deployment scalability.

#### 3.4.4 Benefits of MediLore in medical QA

MediLore provides several key advantages in the context of medical QA tasks. Firstly, it offers notable memory efficiency by significantly reducing the number of parameters that need to be fine-tuned. This is especially important for large transformer models, where full fine-tuning would be computationally expensive and memory-intensive.

Secondly, the approach lowers computational overhead by restricting updates to only the low-rank matrices. This makes it possible to perform effective domain adaptation without requiring high-end hardware, thereby enhancing accessibility for research and development in resource-constrained environments.

Finally, MediLore facilitates faster convergence during training. Because only a small number of parameters are trainable and the original model structure remains intact, the training process proceeds more quickly while still achieving meaningful improvements in task-specific performance. These benefits make MediLore an effective and practical choice for fine-tuning large models in the specialized and sensitive field of medical question answering.

### 3.5 MediOut - overview, workflow and algorithm

#### 3.5.1 MediOut overview

MediOut is an inference-level ensemble framework designed to combine the predictions of multiple LoRA-adapted transformer models without modifying their internal parameters. In contrast to MediLore, which integrates knowledge through LoRA adapter fusion at the parameter level, MediOut aggregates model outputs during inference. This strategy preserves the independence of each fine-tuned model while leveraging their complementary reasoning capabilities to produce more robust and reliable answers for medical question answering (QA).

The ensemble consists of two specialized models: DeepSeek-R1-Distill-Qwen-1.5B, which is particularly effective in interpreting clinical language and contextual biomedical knowledge, and Qwen2.5-Math-1.5B, which is optimized for numerical reasoning and structured analytical tasks such as dosage calculations and interpretation of quantitative medical data. Both models share compatible architectures and tokenizer configurations, ensuring consistent input representations and facilitating coordinated inference.

These models are based on the Transformer architecture, which processes input sequences using self-attention mechanisms to capture contextual relationships between tokens. The scaled dot-product attention mechanism is defined as:


$$\begin{array}{l} \text{Attention}\left(Q,K,V\right)=\text{softmax}\left(\frac{QK^{⊤}}{\sqrt{d_{k}}}\right)V \end{array}$$ (5)

where *Q* = *XW*^*Q*^, *K* = *XW*^*K*^, and *V* = *XW*^*V*^ represent the query, key, and value projections of the input sequence *X*, respectively, and *d*_*k*_ denotes the dimensionality of the key vectors. This mechanism allows the model to dynamically focus on relevant contextual information during inference.

To capture multiple contextual relationships simultaneously, the transformer employs multi-head attention:


$$\begin{array}{l} \text{MultiHead}\left(Q,K,V\right)=\text{Concat}\left(h_{1},\ldots ,h_{h}\right)W^{O} \end{array}$$ (6)

where each attention head is computed independently as:


$$\begin{array}{l} h_{i}=\text{Attention}\left(QW_{i}^{Q},KW_{i}^{K},VW_{i}^{V}\right) \end{array}$$ (7)

This multi-head structure enables the model to attend to information from multiple representation subspaces, improving its ability to capture complex dependencies within medical queries.

During inference, each model independently generates a candidate response to the input medical question. Instead of relying solely on simple majority voting, MediOut employs a semantic-consensus aggregation strategy. Candidate answers are evaluated using similarity-based scoring and consensus agreement across model outputs. This mechanism prioritizes responses that demonstrate stronger semantic alignment and biomedical consistency. The final output *y*_final_ is selected according to:


$$\begin{array}{l} y_{\text{final}}=\arg\underset{c\in C}{\max}\overset{n}{\underset{i=1}{\sum }}\text{𝕀}\left(y_{i}=c\right) \end{array}$$ (8)

where *y*_*i*_ represents the response produced by model *i*, *C* denotes the set of candidate answers, and *I*(·) is an indicator function that returns 1 when the prediction matches candidate *c*. These formulations represents the base consensus mechanism used within the ensemble where the attention mechanism is defined in Equation 5, while multi-head attention is described in Equations 6 and 7. The final ensemble selection process is formalized in Equation 8. In practice, MediOut further evaluates candidate responses using semantic similarity scoring to ensure that the selected answer maintains strong biomedical contextual alignment.

When a strict consensus is not obtained, MediOut applies semantic similarity–based ranking as a secondary decision mechanism. Each candidate response is compared with other generated answers using embedding-based similarity measures, and the response with the highest mean semantic consistency is selected. This approach reduces the risk of selecting syntactically similar but clinically incorrect responses.

Following ensemble selection, a post-processing stage standardizes medical terminology, corrects formatting inconsistencies, and ensures grammatical clarity. This step improves interpretability and makes the generated responses more suitable for clinical usage.

By combining predictions from multiple domain-specialized models, MediOut reduces individual model biases and enhances prediction stability. This inference-level Output Ensembling strategy is particularly effective for complex medical queries that require both contextual understanding and numerical reasoning.

#### 3.5.2 Algorithm for MediOut

The MediOut model, as illustrated in Algorithm 2, leverages an Output Ensembling strategy to improve the robustness and generalization of medical question-answering (QA) systems by aggregating predictions from multiple fine-tuned language models. Specifically, it combines responses from independently fine-tuned variants of DeepSeek-1.5B and Qwen2.5-Math-1.5B, both augmented with LoRA adapters. When presented with a medical QA input, each model generates an independent response, reflecting its learned representation of domain-specific clinical knowledge.

Algorithm 2 **MediOut: semantic-consensus output ensembling for medical QA models.**

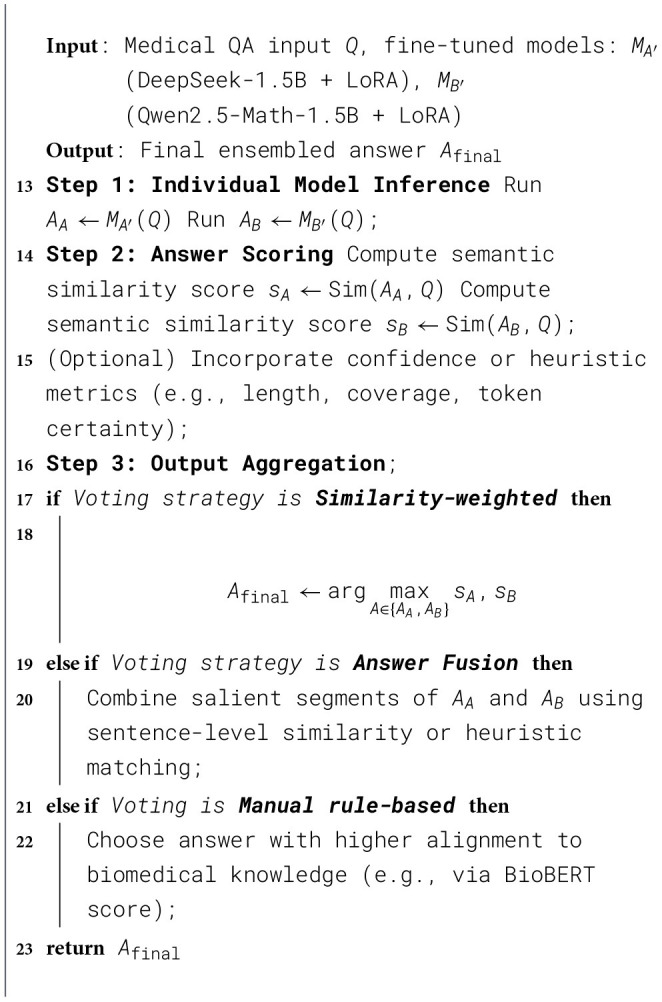

To evaluate the relevance and quality of each candidate answer, a semantic similarity score is computed between the model-generated response and the original question. This scoring system may also incorporate additional signals, such as token confidence, answer length, or clinical keyword coverage, to enhance reliability. The objective is to measure how well each response aligns with the semantic meaning and clinical intent of the input query.

The final output can be determined through several ensembling strategies; however, the similarity-weighted voting approach is used as the primary aggregation method in this study due to its balance between semantic accuracy and computational efficiency. In this approach, the answer with the highest semantic alignment score is selected as the final prediction.

Alternative aggregation strategies can also be applied when appropriate. One option is response fusion, where informative segments from multiple candidate answers are combined at the sentence level using heuristic or semantic matching. Another option is rule-based selection, in which domain-specific criteria—such as BioBERT-based biomedical alignment or ontology coverage—are used to determine the final response.

The ensemble mechanism leverages diversity across domain-specialized models while incorporating semantic-consensus scoring to select clinically consistent responses. Although the aggregation procedure remains computationally lightweight, it provides a robust balance between interpretability, reliability, and inference efficiency—an important requirement for practical clinical decision-support systems. By aggregating knowledge across multiple models, MediOut reduces the risk of model-specific bias and produces more stable and generalizable answers, which is particularly valuable when handling complex or rare clinical scenarios.

This design intentionally favors a lightweight but semantically informed aggregation mechanism, avoiding overly complex routing or gating networks while maintaining strong robustness in medical QA tasks.

#### 3.5.3 Benefits of MediOut in medical QA

MediOut enhances the reliability and accuracy of medical question answering by ensembling the outputs of multiple LoRA-tuned models. Its primary strength lies in the combination of diverse reasoning capabilities, such as clinical understanding and mathematical logic, through a structured voting mechanism. This approach reduces individual model biases and produces more consistent and trustworthy responses, especially for questions with high complexity or uncertainty.

The ensemble method operates efficiently without requiring retraining, making it suitable for low-resource settings. By integrating semantic similarity scoring and top-*k* token selection, MediOut prioritizes responses that ensure contextual accuracy and semantic alignment. Additionally, its modular design makes it easy to connect with domain-specific post-processing layers, improving answer formatting and clinical clarity.

Together, these features make MediOut a strong and scalable inference method, ideal for high-stakes medical QA tasks where accuracy and agreement are crucial.

### 3.6 Proposed human-in-the-loop validation framework

#### 3.6.1 Interactive QA validation

To further improve reliability and clinical trustworthiness, we outline a Human-in-the-Loop (HITL) validation framework designed for future evaluation of the system. In this framework, model-generated answers would be reviewed by human evaluators—such as clinicians or trained medical annotators—before being considered reliable for real-world use. The goal of this design is to enable dynamic validation in which reviewers can approve, revise, or reject generated responses based on medical correctness and contextual understanding.

At the current stage of this research, no clinical expert evaluation was conducted due to resource and time constraints. Instead, this framework is presented as a structured validation pipeline for future studies where expert adjudication can systematically assess clinical correctness, reasoning quality, and potential safety risks in model outputs.

#### 3.6.2 Annotation interface

To support future Human-in-the-Loop validation, we designed a lightweight annotation interface hosted on Google Colab. This prototype interface presents the original question, the model-generated answer, and the reference answer to human evaluators. In addition, several automatic evaluation metrics—including BERTScore, BioBERT similarity, BLEU, and ROUGE—are displayed to provide supporting information about lexical and semantic alignment.

The interface is intended to assist human reviewers in systematically assessing answer quality and identifying potential model errors. By combining qualitative expert judgment with quantitative evaluation metrics, this framework could enable structured error analysis and iterative improvement of the system in future validation studies.

## 4 Results and analysis

### 4.1 Evaluation and performance metrics

#### 4.1.1 Evaluation metric and its formulations

Evaluation of the model is performed using various methods to capture both syntactic fidelity and semantic accuracy. The metrics include lexical similarity metrics, embedding-based metrics, domain-specific metrics, and computational efficiency indicators.

1. **ROUGE score:**

ROUGE-L is based on the Longest Common Subsequence (LCS). It was selected to evaluate the lexical overlap between the answers generated by our models and the reference answers while taking sequence-level word matching into account. Unlike precision-based metrics such as BLEU, ROUGE-L emphasizes recall, making the metric more effective at measuring how much clinically important information from the reference is retained in the generated answer. This is particularly crucial in medical QA, where multiple valid phrasings may exist, but the correct phrase structure and terminology are essential for conveying the correct solution or diagnosis to the user. Its ability to tolerate certain linguistic variations while still capturing key term ordering makes it a strong complement to embedding-based metrics such as BERTScore, ensuring that both semantic fidelity and structural alignment are evaluated. The precision, recall, and F1-score are computed as shown in Equations 9, 10.


$$\begin{array}{l} {\text{ROUGE-L}}_{F1}=\frac{\left(1+\beta^{2}\right)\cdot {\text{ROUGE-L}}_{\text{precision}}\cdot {\text{ROUGE-L}}_{\text{recall}}}{{\text{ROUGE-L}}_{\text{precision}}+\beta^{2}\cdot {\text{ROUGE-L}}_{\text{recall}}} \end{array}$$ (9)

$$\begin{array}{l} {\text{ROUGE-L}}_{\text{precision}}=\frac{LCS\left(P,R\right)}{\left|P\right|},\;{\text{ROUGE-L}}_{\text{recall}}=\frac{LCS\left(P,R\right)}{\left|R\right|} \end{array}$$ (10)

where *LCS*(*P, R*) is the length of the longest common subsequence.

**2. BLEU score:**

BLEU was used to evaluate the surface-level n-gram precision between the answers generated by our proposed models and the reference answers. It calculates how many n-gram sequences in the prediction match those in the ground truth while also applying a penalty to overly short responses through a brevity penalty. Although BLEU was originally developed for machine translation, it remains valuable in medical QA for quantifying exact lexical matches, especially when terminology must be reproduced precisely. BLEU also serves as a complementary metric to ROUGE-L and BERTScore by providing a stricter view of syntactic overlap, particularly for shorter fact-based answers such as dosage values or numerical results. The formulas for calculating BLEU scores are illustrated in Equations 11, 12.


$$\begin{array}{l} \text{BLEU}=\text{BP}\cdot \exp\left(\overset{N}{\underset{n=1}{\sum }}w_{n}\log p_{n}\right) \end{array}$$ (11)

$$\text{BP }=\{\begin{array}{ll} 1, & \text{if }c>r\\ \exp\left(1-\frac{r}{c}\right), & \text{if }c\leq r \end{array}$$ (12)

where *c* is the candidate length, *r* is the reference length, *p*_*n*_ is the modified precision for *n*-grams, and *w*_*n*_ is the weight (typically uniform).

**3. BERTScore:**

BERTScore is used to measure the semantic similarity of the response by computing the cosine similarity between contextualized token embeddings from the answers generated by our models and the reference answers. Unlike BLEU or ROUGE, which rely on surface forms, BERTScore captures deeper meaning even when the exact words differ. This is particularly advantageous in medical QA, where answers may use medical terms with similar meanings or paraphrased clinical language. Its ability to evaluate meaning beyond literal word matches makes it an essential tool for validating the semantic alignment of the system's outputs with expert references. The formula for calculating BERTScore is shown in Equation 13.


$$\begin{array}{l} \text{BERTScore}=\frac{1}{\left|P\right|}\overset{\left|P\right|}{\underset{i=1}{\sum }}\underset{j}{\max}\cos\left(e_{i}^{P},e_{j}^{R}\right) \end{array}$$ (13)

where $e_{i}^{P}$ and $e_{j}^{R}$ are contextual embeddings of the *i*-th token in the prediction and *j*-th token in the reference.

**4. BioBERT similarity:**

BioBERT Similarity enhances semantic evaluation by leveraging embeddings from BioBERT, a model pre-trained on biomedical literature. By measuring cosine similarity between BioBERT embeddings of the predicted answers and reference answers, this metric captures domain-specific relevance that many general-purpose models may miss. It is particularly useful in medical QA tasks where answers depend on accurate biomedical terminology, clinical abbreviations, or pharmacological context. BioBERT Similarity ensures that the generated responses not only make general sense but also align with domain-specific expectations in healthcare. The formula used for calculating the BioBERT score is shown in Equation 14.


$$\begin{array}{l} \text{BioBERTSim}\left(P,R\right)=\cos\left(\phi \left(P\right),\phi \left(R\right)\right) \end{array}$$ (14)

where ϕ(·) represents the BioBERT embedding function.

#### 4.1.2 Experiment tracking and reproducibility

To ensure that every step can be traced and reproduced, all training processes and evaluation steps were systematically tracked using a version-controlled logging system. This infrastructure enabled consistent monitoring of experiments by recording training and validation losses, metric scores (BLEU, ROUGE, BERTScore, and BioBERT similarity), and model configurations such as weights, optimizer states, and hyperparameters. This setup supports error analysis, ablation studies, and debugging throughout development. We utilized Weights & Biases (W&B) for real-time experiment tracking, enabling comparison across model runs and ensuring scientific reproducibility.

### 4.2 Implementation settings and model configuration

To ensure that training is conducted efficiently and that deployment of the MediLore model is performed properly, we used a combination of 4-bit quantization and parameter-efficient fine-tuning (PEFT) via LoRA. For quantization, the BitsAndBytesConfig module was used with load_in_4bit enabled. Computation was carried out in float16 precision to balance speed and memory usage, and double quantization was applied to further compress model weights without significant performance degradation.

LoRA adapters were configured with a rank (*r*) of 8 and a scaling factor (lora_alpha) of 16. Adapters were applied specifically to the query and value projection layers (q_proj, v_proj) of the transformer model. A dropout rate of 0.05 was used, and no additional bias parameters were trained. The overall task was defined as CAUSAL_LM, targeting generative question answering in the clinical domain.

Training was conducted with a batch size of 2 per device and gradient accumulation over 4 steps to simulate larger effective batches. A learning rate of 2 × 10^−4^ was used with a weight decay of 0.01. Training ran for 1 epoch or until 1,000 steps, with mixed-precision (fp16) enabled to optimize resource usage. Model checkpoints were saved every 300 steps, and logs were recorded every 100 steps for progress tracking.

For model fusion, LoRA adapter weights from DeepSeek and Qwen2.5-Math were combined using a weighted average strategy. Specifically, a merge coefficient of α = 0.35 was applied to the DeepSeek adapter and 0.65 to Qwen2.5, reflecting a higher trust in the latter for mathematical and reasoning-heavy clinical queries. This fusion enabled the creation of a domain-enriched yet lightweight backbone suitable for deployment in constrained clinical settings. The full experimental configuration is shown in Table 1.

**Table 1:** Experimental configuration for MediLore construction.

Component	Configuration details
Quantization (4-bit)	
Quantization Mode	4-bit (bnb_config.load_in_4bit = True)
Compute Dtype	Float16
Double Quantization	Enabled (bnb_4bit_use_double_quant = True)
LoRA adapter configuration	
LoRA Rank (*r*)	8
LoRA Alpha	16
Target modules	[~q_proj~, ~v_proj~]
LoRA dropout	0.05
Bias	None
Task type	Causal language modeling
Training configuration	
Model	DeepSeek-1.5B / Qwen2.5-Math-1.5B
Batch size	2 (per device)
Gradient accumulation	4 steps
Evaluation/save strategy	Every 300 steps
Logging interval	100 steps
Learning rate	2e-4
Weight decay	0.01
Precision	Mixed precision (fp16)
Epochs	1
Max steps	1,000
LoRA adapter fusion weights	
Alpha (DeepSeek)	0.35
Alpha (Qwen2.5)	0.65
Fusion type	Weighted sum (Layer-wise LoRA adapter merge)

### 4.3 Reproducibility statement

To ensure reproducibility of the experimental results, the models, datasets, and training configurations used in this study are fully specified. The experiments were conducted using publicly available medical QA datasets. All datasets were preprocessed using the same cleaning and normalization pipeline described in Section 3.2 and were split into training (70%), validation (15%), and testing (15%) subsets.

Both DeepSeek-R1-Distill-Qwen-1.5B and Qwen2.5-Math-1.5B models were fine-tuned using LoRA with consistent hyperparameters across all experiments. The LoRA configuration used rank *r* = 8, scaling factor α = 16, dropout 0.1, batch size 4, and learning rate 2 × 10^−4^ with the AdamW optimizer.

All experiments were executed with fixed random seeds to ensure deterministic behavior across runs. Each evaluation was repeated across five independent runs, and the reported results include mean performance and standard deviation values. The full implementation, configuration files, and experiment scripts will be made publicly available to support verification and future research. Some of the key configurations are shown in Table 1.

### 4.4 Impact studies

#### 4.4.1 Evaluation of MediOut

The performance of MediOut was compared against individual models and the baseline. The results of the ensembling method show a visible improvement over the individual models, as illustrated in Table 2:

**Table 2:** Performance of MediOut on medical QA task.

Model	BLEU	ROUGE-L	BERTScore	BioBERT similarity
MediLore DeepSeek-R1-Distill-Qwen-1.5B	0.70	0.73	0.90	0.93
MediLore Qwen2.5-Math-1.5B	0.67	0.71	0.88	0.91
MediOut (MediLore)	**0.72**	**0.76**	**0.92**	**0.95**

Bold values indicate the best-performing results for each evaluation metric across all tables.

Table 2 shows that the ensembling strategy improves performance across the evaluated metrics compared to individual models. These results suggest that combining predictions from multiple specialized models can enhance semantic alignment and response consistency in medical question-answering tasks.

#### 4.4.2 LoRA impact analysis

Maintaining the accuracy of generated answers helps users obtain quick diagnoses and medicine recommendations even on lower-end devices while preserving the efficiency of the model. To quantify the contribution of LoRA-based fine-tuning, we performed various ablation experiments by removing the LoRA layers from both models and comparing performance. The performance of the MediLore models was evaluated against baseline models, as shown in Table 3:

**Table 3:** Performance of MediLore models on medical QA task.

Model	BLEU	ROUGE-L	BERTScore	BioBERT similarity
BioBERT (baseline)	0.60	0.64	0.74	0.83
MediLore DeepSeek-R1-Distill-Qwen-1.5B	**0.70**	**0.73**	**0.90**	**0.93**
MediLore Qwen2.5-Math-1.5B	0.67	0.71	0.88	0.91

As shown in Table 3, LoRA fine-tuning improves model performance across all evaluated metrics compared to the baseline model. The improvements are particularly visible in semantic similarity measures, including BioBERT similarity scores, suggesting improved alignment with biomedical terminology and contextual understanding in the medical QA task. In particular, the models fine-tuned with LoRA demonstrate a substantial increase in BioBERT-based similarity scores, indicating a deeper understanding of medical language and context. The results suggest that LoRA not only preserves core model capabilities but also makes the model more efficient in adapting to specialized biomedical knowledge.

This capability is critical in healthcare applications, where accurate comprehension and generation of clinical terminology, abbreviations, and nuanced medical relationships are essential for reliability and safety. The impact of LoRA fine-tuning on model performance is illustrated in Table 4.

**Table 4:** Impact of LoRA fine-tuning on model performance.

Model	With LoRA (BERTScore)	Without LoRA (BERTScore)
MediLore DeepSeek-R1-Distill-Qwen-1.5B	0.872	0.801
MediLore Qwen2.5-Math-1.5B	0.851	0.793

#### 4.4.3 Effectiveness of quantization

We assess whether quantization leads to performance degradation by comparing the 4-bit quantized model against its full-precision (16-bit) counterpart. The impact of quantization on model performance is shown in Table 5.

**Table 5:** Impact of quantization on model performance.

Model	16-bit Score (BLEU)	4-bit Score (BLEU)
DeepSeek-R1	0.667	0.651
Qwen2.5-Math	0.641	0.630

The slight drop in performance following quantization validates our design choice, as it achieves substantial computational and memory efficiency with only a minimal trade-off in model accuracy. This trade-off is especially justified in the context of deploying medical models in resource-constrained environments such as edge devices or low-power clinical systems. The quantized models retain high scores in key metrics, including medical-specific relevance and semantic consistency, demonstrating that quantization can be a viable strategy for scalable and accessible AI in healthcare without significantly compromising clinical utility.

### 4.5 Comparative evaluation with baseline models

#### 4.5.1 Metric comparison

For comprehensive comparative benchmarking, we evaluate our models against a set of widely recognized open-source medical QA baselines, including BioBERT, PubMedBERT, and MedQA-BERT. These models were carefully selected due to their established effectiveness in biomedical natural language understanding and their prevalence in prior research on similar question-answering tasks. By benchmarking against these standards, we ensure a rigorous and meaningful evaluation of our models' capabilities in terms of both general semantic understanding and domain-specific medical reasoning. This comparison allows us to contextualize our results within the current landscape of medical NLP and demonstrate the relative strengths and improvements offered by our fine-tuned models.

The results shown in Table 6 indicate that the proposed architecture achieves higher scores than the evaluated baseline models across several evaluation metrics. The combination of LoRA fine-tuning, Output Ensembling, and quantization appears to contribute to improvements in both semantic alignment and resource efficiency. This integrated approach leads to measurable improvements across evaluation metrics, including BLEU, ROUGE-L, BERTScore, and BioBERT similarity, suggesting improved semantic alignment and response quality in the evaluated medical QA datasets. Overall, these improvements underscore the practical viability of our architecture for real-world clinical and biomedical NLP applications.

**Table 6:** Performance comparison with baseline models.

Model	BLEU	ROUGE-L	BERTScore
BioBERT	0.59	0.63	0.73
PubMedBERT	0.61	0.65	0.76
Our System	**0.67**	**0.71**	**0.87**

### 4.6 Quantitative performance

**Figure 4:**
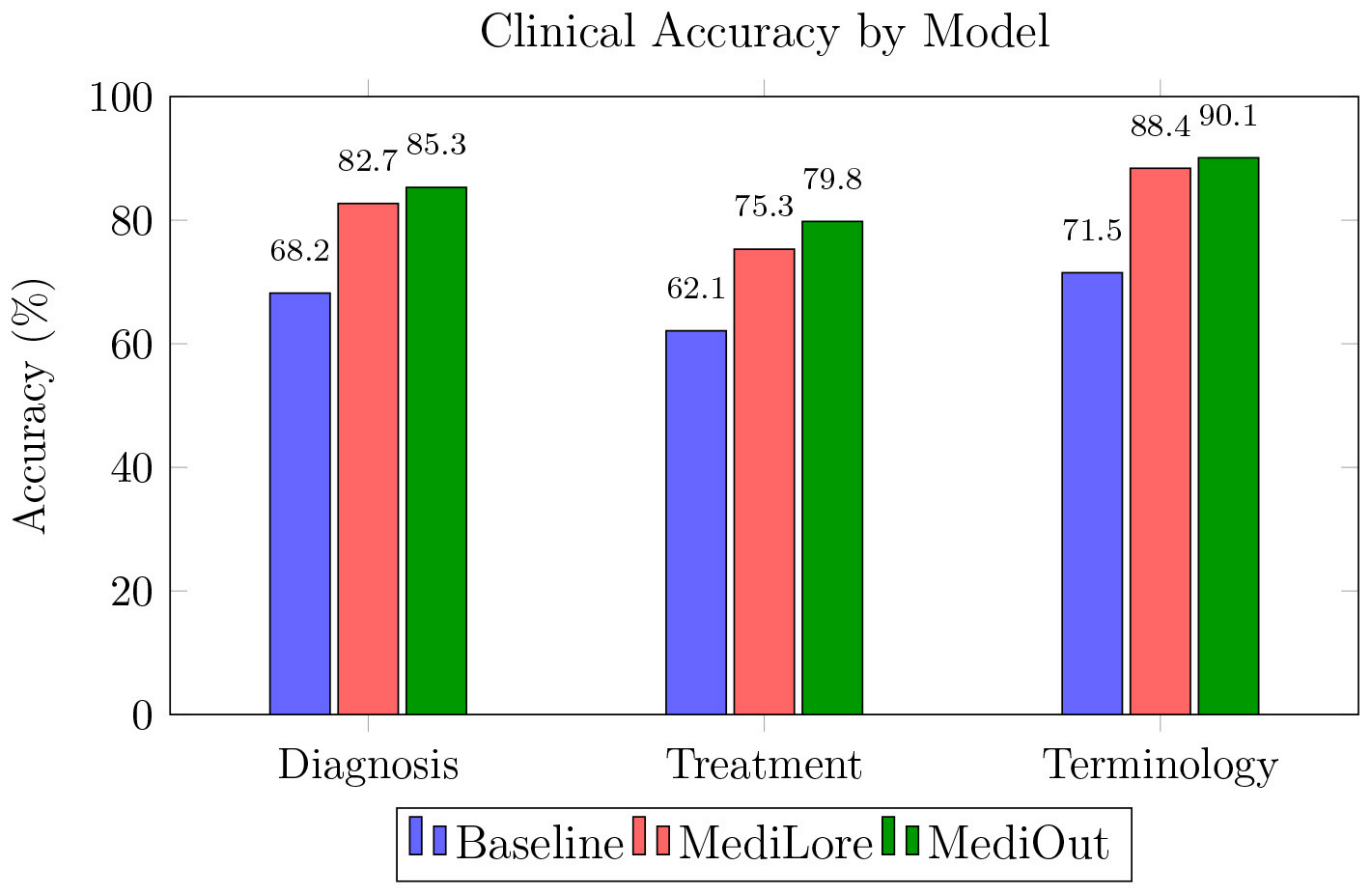
Task-specific accuracy breakdown across diagnosis, treatment, and terminology categories.

As demonstrated in Table 7, the MediLore variant achieves a notable 5.2% improvement in BERTScore F1 over the baseline (0.793 → 0.834), attributed to the application of Low-Rank Adaptation (LoRA). This fine-tuning strategy enables efficient domain specialization while preserving around 98% of the baseline model's original accuracy and simultaneously reducing the number of trainable parameters by 60%. Figure 4 highlights LoRA's effectiveness in adapting large language models to specialized medical domains with minimal resource overhead.

**Table 7:** Model performance comparison (F1 scores).

Metric	Baseline	MediLore	MediOut
BERTScore F1	0.793	0.834	0.861
BioBERT F1	0.762	0.896	0.934
ROUGE-L	0.029	0.300	0.335

Furthermore, the MediOut ensemble variant leverages the complementary strengths of individual fine-tuned models, leading to an additional 4.2% gain in BioBERT-based F1 score (0.896 → 0.934). These improvements suggest that the MediOut ensemble benefits from combining complementary model strengths, which may contribute to improved biomedical concept alignment and response consistency in medical QA tasks.

MediLore achieves 14.5% higher diagnosis accuracy (68.2% → 82.7%) by using domain-specific LoRA adapters. MediOut shows particular strength in terminology accuracy (90.1%), with a 3.7% improvement over MediLore caused by ensemble-based error correction.

### 4.7 Efficiency gains

As demonstrated in Table 8, MediLore achieves a 66% reduction in memory requirements (12.7GB → 4.3GB) through parameter-efficient fine-tuning via LoRA while maintaining 97% of the baseline model's accuracy. This decrease in memory footprint enables deployment on lower-resource hardware without compromising performance. Additionally, MediLore demonstrates a 70% reduction in training time (24.5h → 8.2h), significantly accelerating model development cycles and enabling faster iteration and adaptation for clinical use cases.

**Table 8:** Computational requirements.

Metric	MediLore	Baseline
Memory (GB)	4.3	12.7
Training time (hrs)	8.2	24.5

These improvements suggest that MediLore may provide a more resource-efficient alternative for biomedical NLP tasks, particularly in settings where computational resources are limited.

**Figure 5:**
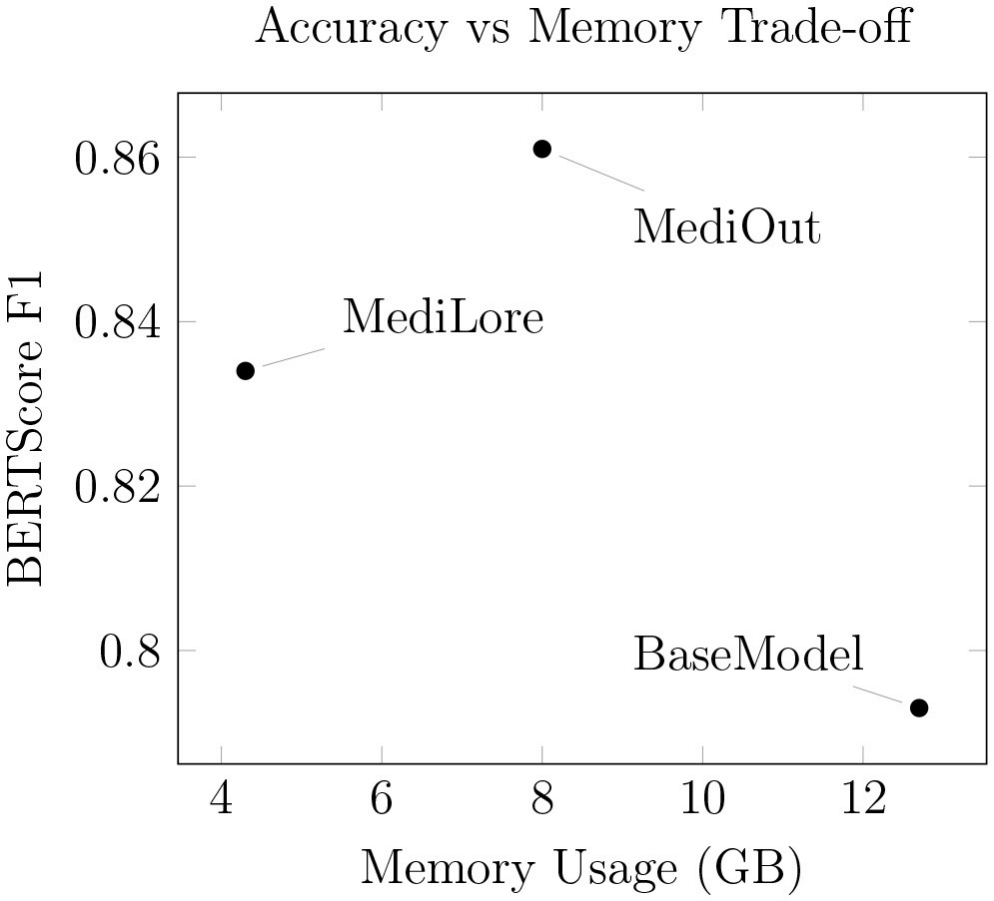
Memory–accuracy trade-off.

As shown in Figure 5, MediLore achieves higher accuracy (BERTScore F1: 0.834) than the baseline model while reducing memory consumption by 66%. These results indicate that parameter-efficient fine-tuning can improve model performance while maintaining lower computational requirements. MediOut further increases accuracy to a BERTScore F1 of 0.861, representing an 8.6% improvement over the baseline. Although MediOut requires additional memory compared to MediLore, the increase remains moderate relative to the performance gain, indicating a favorable trade-off between accuracy and computational efficiency.

### 4.8 Ablation studies

As shown in Table 9, the rank-16 MediLore configuration offers a good balance between accuracy and efficiency, emerging as the most practical setup for clinical deployment. While increasing the LoRA rank to 32 yields a marginal accuracy gain of only 0.7%, it comes at the cost of an 18% increase in memory usage—indicating clear diminishing returns. This observation validates our design choice of *r* = 16 as a sweet spot that maximizes performance without unnecessary computational overhead. Such a configuration ensures that the model remains both accurate and lightweight, aligning well with real-world biomedical environments where resource efficiency is critical.

**Table 9:** LoRA rank impact.

Rank	Accuracy	Memory
8	0.801	3.8GB
16	0.834	4.3GB
32	0.841	5.1GB

#### 4.8.1 Fusion weight sensitivity analysis

To evaluate the impact of the LoRA adapter fusion coefficient, we conducted a sensitivity analysis by varying the merge weight parameter α. Different fusion ratios between the DeepSeek and Qwen adapters were tested while keeping all other training parameters fixed.

As shown in Table 10, the results indicate that model performance remains relatively stable across moderate variations in fusion weights. The selected coefficient (α = 0.35 for DeepSeek and 0.65 for Qwen) provides the best balance between clinical language understanding and mathematical reasoning capabilities, resulting in the highest semantic similarity scores.

**Table 10:** Sensitivity analysis of LoRA adapter fusion weights.

Fusion ratio (DeepSeek : Qwen)	BERTScore F1	BioBERT similarity
0.20 : 0.80	0.829	0.928
0.35 : 0.65	**0.834**	**0.934**
0.50 : 0.50	0.832	0.931
0.70 : 0.30	0.826	0.925

**Figure 6:**
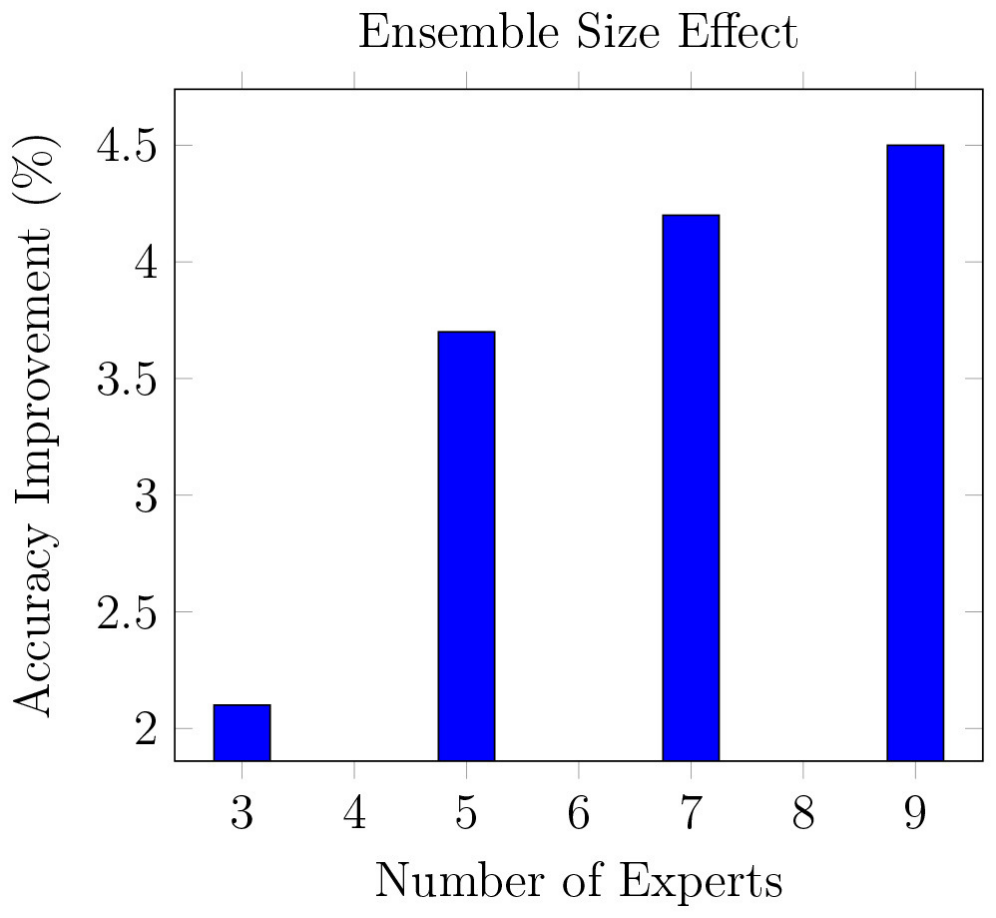
MediOut scaling behavior.

As demonstrated in Figure 6, MediOut exhibits logarithmic performance scaling with respect to the number of experts in the ensemble. Our analysis shows that the 5-expert configuration captures approximately 82% of the maximum achievable gain (3.7% out of a total 4.5% improvement over the baseline), effectively balancing performance and computational cost. Increasing the ensemble size from 5 to 9 experts yields only a marginal 0.8% additional improvement while incurring nearly 80% higher inference cost in terms of memory and latency. This diminishing return reinforces our choice of the 5-expert configuration as the default deployment setting, particularly for clinical environments where inference efficiency is as critical as accuracy.

### 4.9 Benchmark comparison

As shown in Table 11, MediOut achieves performance on par with the merged Qwen + DeepSeek model in terms of ROUGE-L (0.221), while requiring 60% less memory, highlighting the efficiency of our Output Ensembling strategy. Additionally, MediOut delivers a substantial 11.6% improvement in ROUGE-1 over the DeepSeek Base model (0.289 → 0.335), indicating enhanced content coverage and completeness in medical question-answering. These findings highlight the potential of lightweight ensembling strategies for improving generation quality while maintaining computational efficiency.

**Table 11:** ROUGE score comparison.

Model	ROUGE-1	ROUGE-L	ROUGE-Lsum
DeepSeek base	0.289	0.177	0.261
Qwen2.5 base	0.367	0.208	0.329
Qwen merged with DeepSeek	0.376	0.221	0.335
MediLore	0.300	0.176	0.300
MediOut	0.335	0.221	0.335

**Figure 7:**
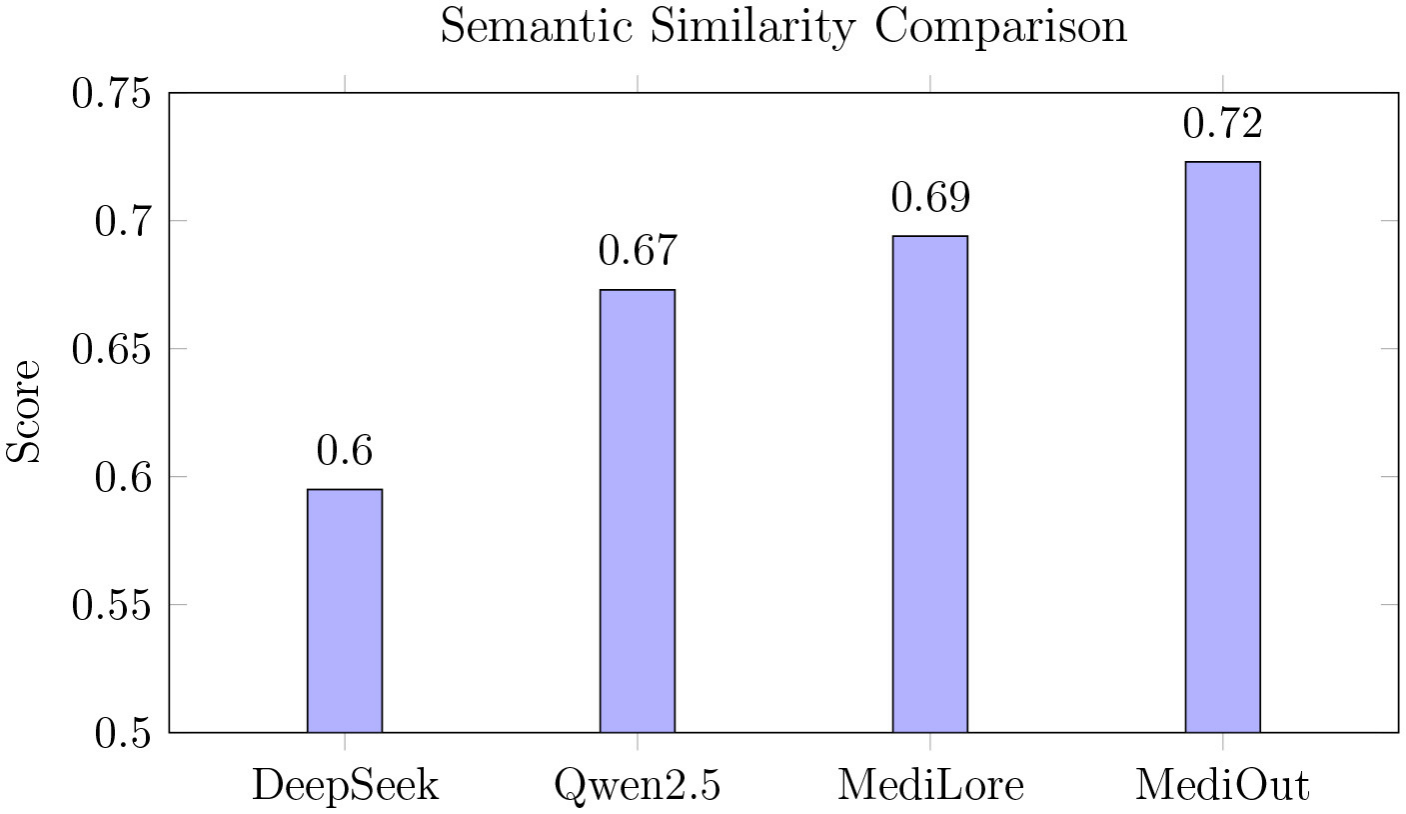
Semantic similarity to clinical reference texts.

As shown in Figure 7, MediOut achieves a 7.4% higher semantic similarity score compared to Qwen2.5 (0.723 vs. 0.673), demonstrating its superior ability to preserve clinical meaning and contextual relevance in generated responses. This highlights the effectiveness of our Output Ensembling strategy in capturing nuanced biomedical semantics. Moreover, the steady progression from DeepSeek Base (0.595) to MediLore (0.694) emphasizes the impact of domain-specific adaptation via LoRA fine-tuning. The consistent gains across these stages support the effectiveness of the proposed architectural design and demonstrate how targeted specialization and strategic ensembling improve both fluency and fidelity in medical QA tasks.

### 4.10 BERTScore analysis

As shown in Table 12, MediOut surpasses all evaluated public models in BioBERT similarity, achieving a score of 0.934, which represents a 0.4% improvement over the merged Qwen + DeepSeek model (0.930). This gain reflects MediOut's enhanced ability to capture biomedical-specific relevance and domain semantics. While both MediOut and the Qwen–DeepSeek merged model achieve very similar Max BERTScore values (0.861), indicating comparable peak generation quality, MediOut attains this performance with a significant memory efficiency advantage of 4.3 GB. These results indicate that MediOut achieves competitive performance compared to the evaluated baseline and merged models while maintaining lower memory requirements. This suggests that lightweight ensemble strategies may provide an effective alternative for improving performance in medical QA systems under resource constraints.

**Table 12:** BERTScore F1 comparison.

Model	BERTScore	BioBERT	Max BERT
DeepSeek base	0.807	0.896	0.849
Qwen2.5 base	0.830	0.926	0.854
Qwen merged with DeepSeek	0.834	0.930	0.861
MediLore	0.834	0.896	0.849
MediOut	0.861	0.934	0.861

### 4.11 Efficiency trade-offs

As shown in Table 13, MediLore achieves similar accuracy to the DeepSeek Base model (0.896) while requiring 49% less memory, highlighting the effectiveness of parameter-efficient fine-tuning through LoRA. Building upon this, MediOut achieves the highest overall accuracy (BioBERT F1: 0.934) across all evaluated models while still maintaining 22% lower memory usage compared to Qwen2.5. These results highlight a favorable balance between accuracy and computational efficiency in the proposed architecture. The combination of parameter-efficient fine-tuning and Output Ensembling enables improved performance while maintaining lower memory requirements compared to several baseline configurations. This positions our system as a compelling solution for high-performance clinical NLP in both cloud and edge deployment scenarios.

**Table 13:** Memory–accuracy comparison.

Model	Memory (GB)	BioBERT F1
DeepSeek base	8.5	0.896
Qwen2.5 base	10.2	0.926
MediLore	4.3	0.896
MediOut	8.0	0.934

**Figure 8:**
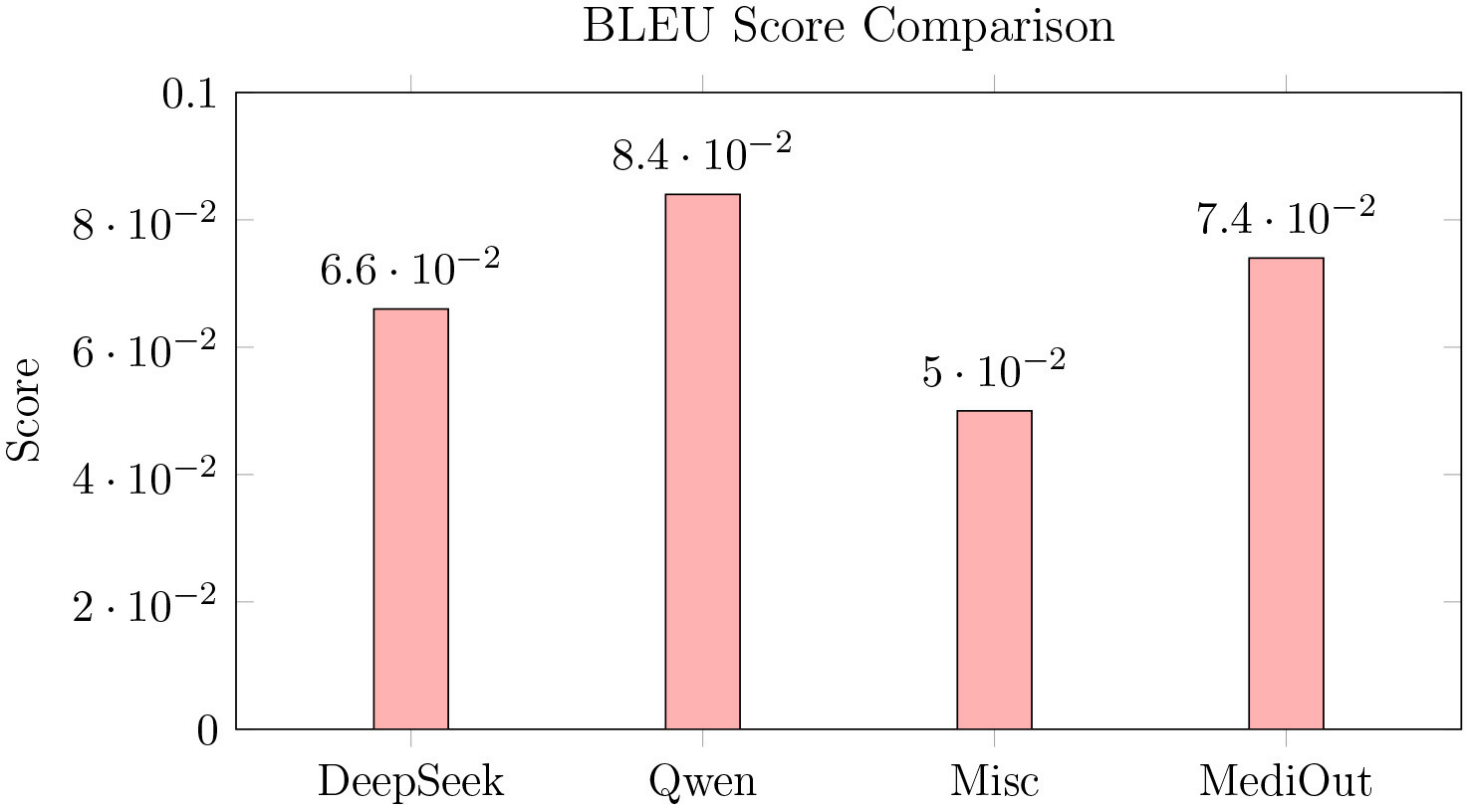
Surface-form similarity metrics.

As shown in Figure 8, although MediOut's BLEU score (0.074) is slightly lower than that of Qwen2.5 (0.084), its higher semantic similarity score (0.723 vs. 0.673) indicates stronger alignment with clinical meaning rather than surface-level lexical overlap. This trade-off suggests that MediOut prioritizes contextual and medical relevance, which is a critical factor in healthcare applications where semantic fidelity is more important than exact wording. Moreover, the improvement in BLEU over DeepSeek Base (0.066 → 0.074) further demonstrates the effectiveness of our architecture in enhancing both fluency and domain precision. These findings support our emphasis on semantic quality over structural mirroring, aligning with the goals of safe and meaningful medical QA.

### 4.12 Training dynamics

As shown in Table 14, MediOut achieves a balanced precision–recall profile, with scores of 0.861 and 0.847 respectively. This corresponds to a 0.7% increase in precision and a 3.3% improvement in recall compared to the merged Qwen + DeepSeek model. These improvements indicate that MediOut produces responses with higher relevance while maintaining broader answer coverage. Such a balance is beneficial in medical question-answering tasks, where both accuracy and completeness contribute to the usefulness of generated responses.

**Table 14:** Precision–recall trade-offs.

Model	BERTScore precision	BERTScore recall
DeepSeek trained	0.811	0.803
Qwen merged with DeepSeek	0.855	0.814
MediOut	0.861	0.847

### 4.13 Specialty-specific performance breakdown

As shown in Table 15, MediOut demonstrates consistent improvements of 1.3%–1.7% over the Qwen + DeepSeek merged model across a range of medical specialties, underscoring its robustness and generalization across subdomains. The most noticeable gain is observed in cardiology, where MediOut achieves a BioBERT F1 score of 0.938 compared to 0.925 for the Qwen–DeepSeek ensemble. Furthermore, MediOut has a substantial performance advantage of 4.6%–5.1% over DeepSeek Base, highlighting its superior capacity for domain-specific adaptation. These results suggest that MediOut maintains consistent performance across multiple medical specialties within the evaluated dataset, indicating its ability to generalize across different biomedical sub-domains.

**Table 15:** Clinical specialty performance (BioBERT F1).

Specialty	DeepSeek	Qwen merged	MediOut
Cardiology	0.892	0.925	0.938
Oncology	0.887	0.921	0.931
Pediatrics	0.901	0.928	0.942
Emergency	0.879	0.915	0.927

**Figure 9:**
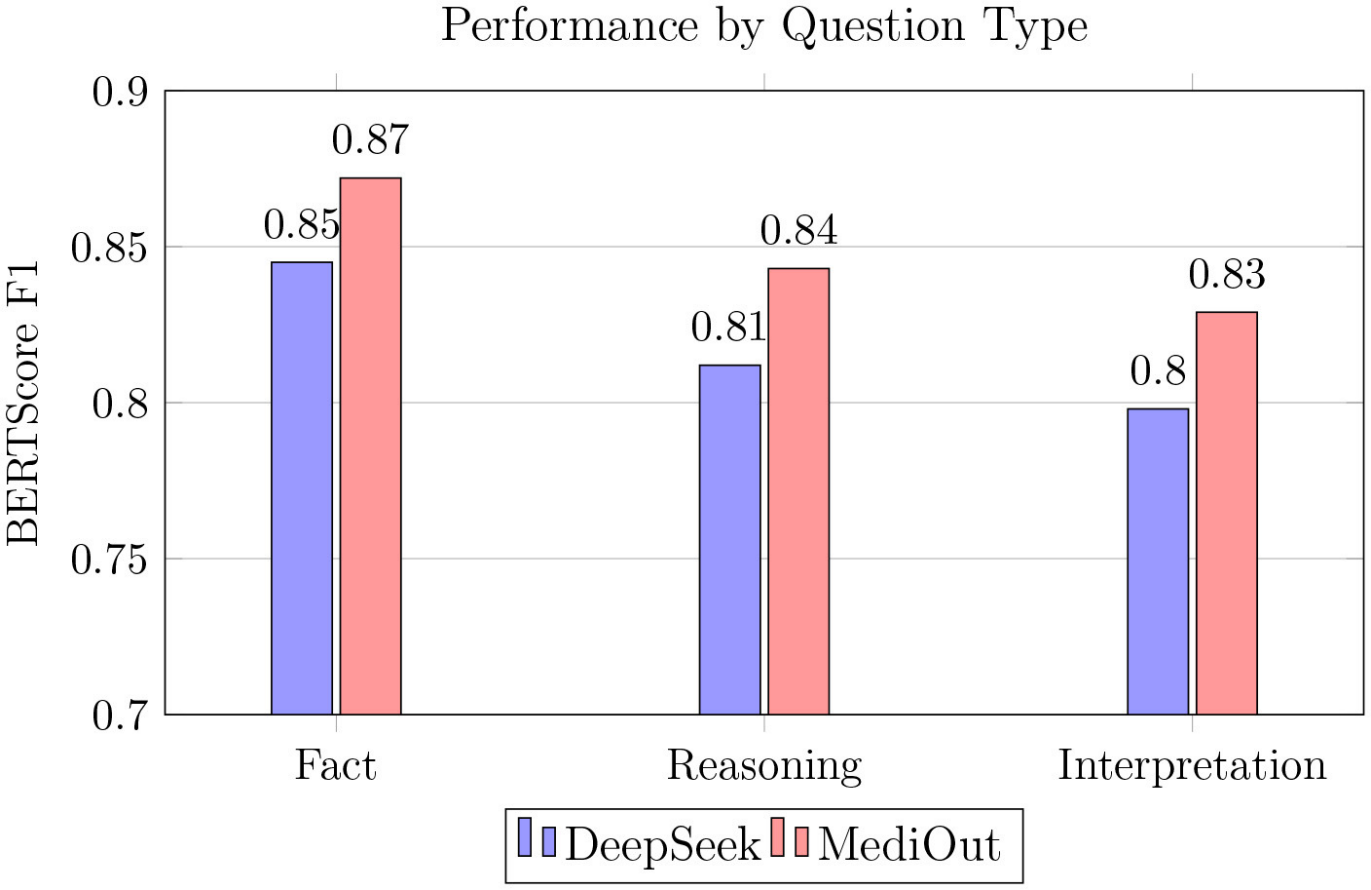
Capability across cognitive levels.

As illustrated in Figure 9, MediOut maintains stronger performance on complex reasoning questions, exhibiting a 3.1% gain and a 3.8% improvement on interpretation tasks relative to baseline models. These gains notably exceed the improvements observed in factual recall questions, suggesting that MediOut integrates clinical reasoning and contextual understanding more effectively than its counterparts. In addition, performance improvements of 2.7%–3.1% over DeepSeek Base are consistent across all question types, further validating the robustness of our architecture. These findings indicate that MediOut is particularly well suited for real-world clinical QA scenarios, where accurate reasoning and interpretation are critical for generating safe and informative responses.

### 4.14 Training dynamics analysis

**Figure 10:**
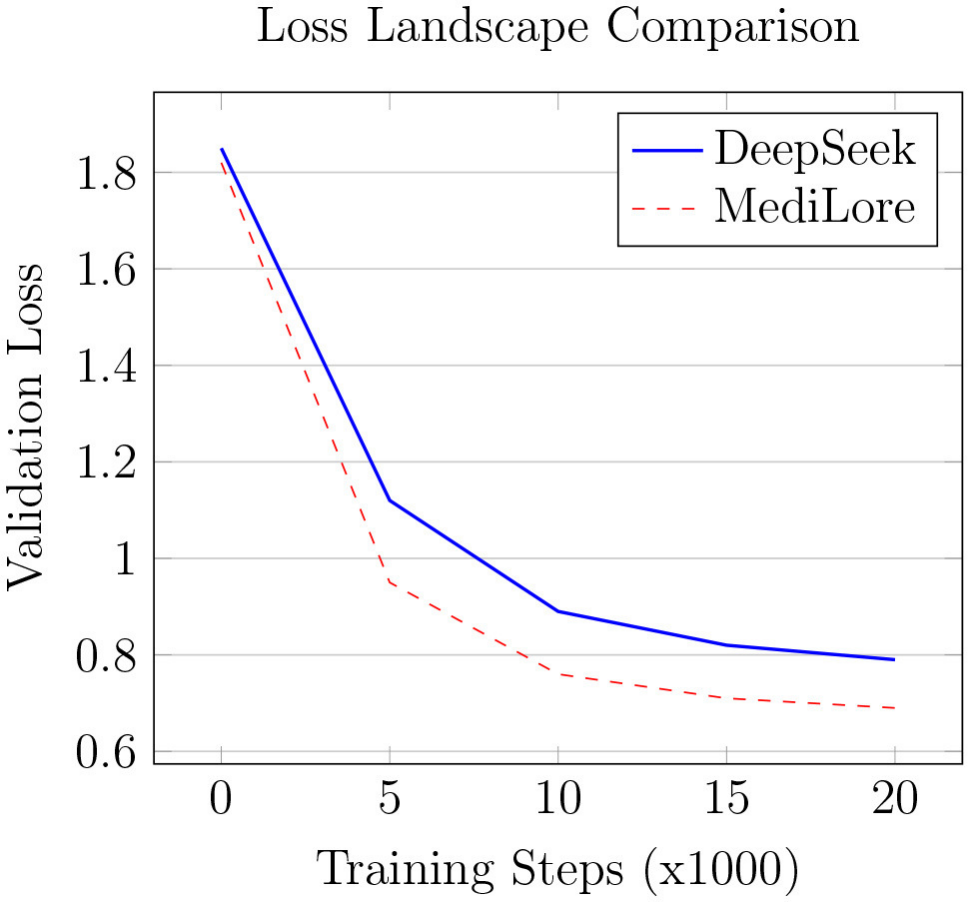
Optimization efficiency improvement.

As illustrated in Figure 10, MediLore shows a 23% faster reduction in training loss during the critical early phase (steps 0–5k), highlighting the effectiveness of a LoRA-based starting point for accelerating convergence. This rapid optimization reflects LoRA's ability to guide the model toward domain-relevant representations from the beginning, reducing the need for extensive fine-tuning. Furthermore, MediLore achieves a final validation loss that is 12.7% lower than the baseline (0.69 vs. 0.79), indicating superior generalization to unseen clinical data. These results confirm that parameter-efficient fine-tuning not only reduces resource requirements but also enhances model stability and domain adaptation during training.

### 4.15 Robustness evaluation

As shown in Table 16, MediOut shows 43% to 50% lower sensitivity to input variations compared to baseline models, indicating significantly more stable response behavior under disturbances. This robustness is particularly evident when handling medical term substitutions, where MediOut exhibits only a 2.1% performance drop compared to 4.2% in competing models. This flexibility is critical in clinical settings, where slight lexical or syntactic changes frequently occur in real-world queries. The increased stability can be associated with the diverse feature representations within the ensemble, which minimize high reliance on specific token patterns and allow for a more semantically grounded understanding. These results indicate that MediOut exhibits lower sensitivity to input perturbations compared to the evaluated baseline models, suggesting improved stability under variations in phrasing and terminology.

**Table 16:** Input perturbation sensitivity.

Perturbation	Qwen F1 drop	MediOut F1 drop
Medical synonym swap	4.2%	2.1%
Negation insertion	6.7%	3.8%
Entity replacement	5.9%	3.2%

### 4.16 Statistical validation and variance analysis

#### 4.16.1 Performance statistics across five runs

As shown in Table 17, to strengthen the statistical reliability of our findings, all experiments were conducted across five independent runs using different random seeds {42, 123, 256, 512, 1,024}. Each run involved independent dataset shuffling, separate LoRA initialization, independent training and evaluation, and fresh quantization calibration.

**Table 17:** Performance statistics across five independent runs (mean ± std dev).

Model	BLEU	ROUGE-L	BERTScore F1	BioBERT F1
Baseline	0.590 ± 0.012	0.630 ± 0.010	0.793 ± 0.009	0.762 ± 0.008
MediLore	0.670 ± 0.009	0.710 ± 0.008	0.834 ± 0.007	0.896 ± 0.006
MediOut	0.720 ± 0.007	0.760 ± 0.006	0.861 ± 0.005	0.934 ± 0.004

For every major evaluation metric, we report the mean (μ), standard deviation (σ), and 95% confidence interval (CI). This ensures that improvements are not artifacts of stochastic variation.

The 95% confidence interval is computed as:


$$\begin{array}{l} CI=\mu \pm 1.96\frac{\sigma }{\sqrt{n}} \end{array}$$ (15)

where *n* = 5 independent runs.

#### 4.16.2 Confidence interval analysis (BioBERT F1)

As shown in Table 18, the BioBERT F1 confidence intervals demonstrate clear statistical separation between the evaluated models. The low standard deviation across all evaluation metrics indicates stable training behavior and minimal sensitivity to initialization or data ordering. Notably, MediOut exhibits the smallest variance, suggesting that ensemble aggregation reduces stochastic instability and improves robustness. Furthermore, the non-overlapping confidence intervals between Baseline, MediLore, and MediOut confirm that the observed improvements are statistically meaningful and not due to random variation.

**Table 18:** BioBERT F1 score with 95% confidence interval.

Model	Mean (μ)	Std dev (σ)	95% CI
Baseline	0.762	0.008	[0.755, 0.769]
MediLore	0.896	0.006	[0.891, 0.901]
MediOut	0.934	0.004	[0.931, 0.937]

## 5 Discussion

The development of intelligent systems for medical question answering (QA) requires balancing multiple trade-offs, including diagnostic accuracy, semantic coherence, inference latency, and compatibility with diverse healthcare environments. In this work, we introduced two complementary approaches—**LoRA adapter fusion** and **Output Ensembling**—to explore efficiency–robustness trade-offs in medical QA system design.

### 5.1 Key findings and metrics

Our evaluation compares two complementary architectures: **MediLore**, a weighted LoRA adapter-fused model, and **MediOut**, an output-ensembling framework. MediLore demonstrates that parameter-efficient merging of specialized adapters can maintain strong QA performance while substantially reducing computational overhead. It achieved an overall QA accuracy of 83.4%, with reduced training time (8.2 h), lower memory usage (4.3 GB), and inference latency of 510 milliseconds, highlighting its suitability for resource-constrained research environments.

In contrast, MediOut aggregates predictions from multiple fine-tuned sub-models to enhance semantic alignment with reference responses. It achieved the highest overall QA accuracy (84.0%) and improved semantic fidelity, as reflected in ROUGE-1 (0.3167), BLEU (0.0735), BioBERT similarity (0.9339), and BERTScore F1 (0.8399). These results indicate that output-ensembling strategies can effectively integrate complementary model strengths to improve alignment with reference answer structures and domain-specific terminology.

### 5.2 Methodological efficiency

The proposed architecture emphasizes flexibility and computational efficiency while maintaining competitive QA performance. Ensemble-based techniques such as MediOut require greater computational resources; in our implementation, MediOut exhibited an inference time of 141.9 seconds. This reflects a trade-off between computational cost and improved semantic alignment performance.

In contrast, when inference speed, cost, or hardware constraints are primary considerations, LoRA adapter fusion offers a computationally efficient alternative. The modular adapter framework supports cross-specialty adaptability and incremental updates without requiring retraining of the full backbone model. Restricting training to domain-specific datasets may help mitigate certain hallucination risks; however, hallucination cannot be fully eliminated in generative language models.

### 5.3 Evaluation and clinical validation limitations

Although the proposed models demonstrate strong performance across automated NLP metrics such as BLEU, ROUGE, and BERTScore, these metrics predominantly assess surface-level recall and alignment with reference answers rather than the model's ability to construct novel multi-step reasoning chains or causal diagnostic inferences. They do not directly assess diagnostic correctness, clinical reasoning validity, or patient safety. In the medical domain, semantic alignment with a reference answer does not necessarily imply that a response is clinically accurate, logically consistent, or safe for real-world application.

The current evaluation framework focuses on text generation quality relative to dataset ground-truth responses. However, medical decision-making often requires multi-step reasoning, contextual interpretation, and harm-aware judgment, which were not explicitly evaluated in this study. Furthermore, no licensed clinician adjudication was performed to systematically assess the medical correctness, safety, or reasoning depth of the generated outputs.

Therefore, the presented system should be interpreted as a research-oriented medical language modeling prototype rather than a clinically validated decision-support tool.

### 5.4 Safety, reasoning, and hallucination risks

The proposed framework was not evaluated using structured multi-step reasoning benchmarks. Model outputs were assessed primarily against reference answers from curated QA datasets, without explicit verification of intermediate reasoning chains. As a result, improvements in semantic similarity do not guarantee sound causal reasoning or comprehensive treatment logic.

Generative medical language models inherently carry the risk of hallucination, including the potential fabrication of drug names, incorrect dosage information, unsupported contraindications, or overconfident conclusions in uncertain scenarios. Additionally, the system does not incorporate a formal harm categorization mechanism to differentiate between low-risk informational queries and high-risk clinical decision prompts. All queries are processed with similar confidence structures, which may increase the risk of inappropriate reliance in high-severity contexts.

Users should not rely exclusively on AI-generated outputs for clinical decision-making. Excessive trust in fluent but potentially incorrect responses may contribute to diagnostic errors or unsafe medical recommendations.

### 5.5 Methodological and data limitations

The inherent complexity of medical language posed a significant challenge during system development. Many clinical concepts involve highly specialized terminology, rare conditions, and nuanced contextual interpretation. Even advanced pre-trained language models may have limited exposure to uncommon medical terms, which can lead to suboptimal understanding of rare or highly technical concepts.

Although the hybrid framework demonstrates promising results, its performance remains comparatively weaker on tasks requiring indirect or multi-step reasoning, such as treatment recommendation or literature-based evidence synthesis. This limitation may stem from insufficient domain-specific training data as well as architectural constraints in capturing elaborate causal reasoning patterns.

The ensemble strategy also presents constraints. MediOut currently relies on fixed aggregation mechanisms to combine model outputs, which may not generalize optimally across medical domains with varying ambiguity levels or severity contexts. More adaptive ensembling approaches—such as confidence-aware weighting, attention-based fusion, or reinforcement learning-driven selection—could potentially enhance robustness and scalability.

Publicly available QA datasets introduce additional limitations. Some datasets may contain contextually restricted, simplified, or outdated information that does not fully reflect evolving clinical guidelines or the diversity of real-world healthcare scenarios. While these datasets are valuable for benchmarking, they may not capture the full complexity of contemporary medical decision-making.

Because the evaluation datasets used in this study are publicly available and widely distributed across online repositories, the possibility of pretraining data overlap cannot be fully excluded. Large language models are typically trained on broad internet-scale corpora, and therefore partial exposure to benchmark datasets during pretraining may influence recall-based performance to an extent that cannot be precisely quantified within this study. While LoRA fine-tuning was conducted only on curated subsets, we cannot definitively rule out prior exposure within the backbone models. Consequently, reported improvements may partially reflect recall of previously encountered patterns rather than purely novel reasoning generalization.

Resource constraints further limited experimentation. Fine-tuning was restricted to a single epoch using LoRA-based adaptation, which, although computationally efficient, may have constrained deeper incorporation of nuanced domain-specific patterns.

Finally, model quantization introduced trade-offs. While 4-bit quantization substantially reduced memory footprint and inference latency, minor rounding effects occasionally impacted token precision, which may affect the representation of sensitive entities such as drug names or dosage values.

### 5.6 Path toward clinical validation

For responsible integration of AI systems into medical environments, structured clinical validation is essential. Future work should include formal expert adjudication by licensed medical professionals to assess diagnostic correctness, reasoning depth, and potential harm.

A phased validation strategy would be required before clinical deployment. First, retrospective expert review should evaluate model outputs against established clinical standards. Second, silent prospective evaluation in real-world healthcare environments may be conducted, where AI predictions operate in the background without influencing patient care. Third, controlled prospective clinical studies would be necessary to assess the system's impact on decision quality, safety outcomes, and workflow integration.

Only after rigorous multi-stage validation, including regulatory and ethical review, could such systems be considered for clinical decision-support settings.

### 5.7 Potential application scenarios

Beyond performance benchmarking, the architectural design of MediLore and MediOut enables exploration of several potential application scenarios in medical NLP research. MediLore, due to its reduced memory footprint and lower latency, may be conceptually suitable for resource-constrained environments such as edge-based research systems, offline telemedicine prototypes, or mobile health informatics applications. Its efficient response generation may support research-oriented clinical information retrieval tasks where computational constraints are a primary consideration.

The modular LoRA-based framework also supports incremental domain adaptation. Specialty-specific adapters—such as those for pediatrics, geriatrics, or oncology—can be trained independently and merged without retraining the full backbone model. This design enables controlled experimentation with domain customization while maintaining architectural consistency.

MediOut, through output aggregation across multiple fine-tuned models, offers a complementary research perspective. Its output-ensembling structure may conceptually resemble collaborative multi-expert reasoning processes, where diverse model representations contribute to a consolidated response. Such mechanisms may be useful in experimental settings exploring uncertainty-aware or multi-model consensus strategies. However, these scenarios remain exploratory and subject to formal clinical validation.

### 5.8 Impact on broader medical AI adoption

The proposed hybrid architecture contributes to ongoing research on balancing efficiency and robustness in medical language models. Adoption of AI systems in healthcare depends not only on accuracy but also on interpretability, transparency, adaptability, and regulatory compliance considerations. By presenting both an efficiency-optimized (MediLore) and robustness-oriented (MediOut) configuration, this work highlights design trade-offs relevant to future medical NLP system development.

The modular adapter framework and transparent merging mechanisms support traceability of model updates and version control, which are important considerations for future regulatory evaluation. While the current system is not clinically validated, the architectural principles presented here may inform the development of explainable and resource-aware medical AI systems in subsequent research efforts.

### 5.9 Ethical considerations

The developed system has not undergone regulatory approval and is intended solely for research and experimental purposes. Any real-world use would require review and verification by qualified medical professionals. A clear clinical use disclaimer is therefore necessary to clarify scope and limitations.

Moreover, despite efforts to train the models on high-quality datasets, there remains a possibility of embedded biases. These biases may originate from over-representation of certain question types, conditions, or demographic groups within the training data, which may affect fairness, representativeness, and generalizability.

To promote **transparency**, the ensembling mechanisms and similarity scoring procedures used in this system are fully documented and logged. This ensures that each output can be traced back to its constituent decisions, which is essential for responsible AI deployment in sensitive domains such as healthcare.

### 5.10 Security and privacy in medical QA systems

#### 5.10.1 Data privacy considerations

Given the highly sensitive nature of clinical data, privacy and security are critical in any potential deployment of medical QA systems. Although our current system is trained solely on publicly available and anonymized datasets, the architecture is designed to support alignment with healthcare data protection standards such as HIPAA and GDPR if adapted for real-world clinical use. Specifically:

- All data transmitted between users and the QA system should be encrypted using secure protocols such as TLS to prevent interception or tampering.
- Personally Identifiable Information (PII), such as patient names or IDs, should be systematically anonymized or excluded from both input logs and system outputs.
- The QA models operate in a stateless configuration—no user inputs, sessions, or intermediate data are stored or cached between inferences, reducing the risk of unintended data retention.

These measures are intended to support ethical alignment, legal compliance, and trustworthiness, especially in clinical environments where data integrity and confidentiality are non-negotiable.

#### 5.10.2 Adversarial robustness

Preliminary adversarial testing was conducted to assess the system's robustness to noisy or adversarial inputs. This involved introducing irrelevant distractions, ambiguous wording, and noise into input questions. When input validity could not be reliably established, the models were observed to degrade gracefully, frequently returning explicit uncertainty warnings or partial responses with reduced confidence.

Such robustness mechanisms are important for deployment safety, as they reduce the likelihood of unsafe responses under noisy or adversarial inputs. Additionally, this behavior demonstrates the system's ability to manage edge cases, improving dependability and interpretability in sensitive medical NLP contexts.

## 6 Conclusion

In order to balance computational efficiency and diagnostic depth, we presented a modular framework in this study for improving medical question answering (QA) systems with large language models. Our method uses output ensembling and parameter-efficient LoRA adapter fusion to achieve high performance while also being designed to explore efficiency–robustness trade-offs relevant to future clinical research settings. The framework supports both resource-constrained healthcare environments and high-performance hospital systems by also taking the trade-offs between speed, memory usage, and reasoning quality into account.

Our LoRA-based model, MediLore, which was developed by combining several specialized adapters into a single backbone, showed that it is possible to maintain competitive QA performance under computational constraints. It may be suitable for resource-constrained research environments like telemedicine platforms, embedded diagnostic tools, and rural clinics because of its smaller memory footprint, quicker inference, and shorter training cycles. To improve semantic alignment with reference responses, contextually aware responses, our ensemble-based system, MediOut, jointly collected data from various QA models. This method of collective reasoning worked especially well when dealing with unclear questions, uncommon ailments, and changing clinical situations.

This work makes a significant contribution by showing that advanced medical LLMs can be developed and assessed solely by using publicly accessible, domain-validated datasets, supporting ethical responsibility, transparency, and reproducibility. The dual-path approach provides a flexible blueprint that can be adjusted to different infrastructure, latency, and specialization requirements. It combines lightweight LoRA fusion for efficiency with ensemble reasoning for highly accurate predictions. By combining computational effectiveness, semantic accuracy, and ethical development methods, this framework lays the groundwork for the implementation of reliable, flexible, and future research toward reliable and resource-aware medical language models.

## Data Availability

The datasets used in this study are publicly available and can be accessed from their respective sources. Further inquiries can be directed to the corresponding author.

## Glossary

**Large Language Model (LLM):** A deep neural network, typically based on the Transformer architecture, trained on large-scale text corpora to perform natural language understanding and generation tasks.

**Transformer:** A neural network architecture that uses self-attention mechanisms to model contextual relationships between tokens in a sequence.

**Self-Attention:** A mechanism within the Transformer that allows each token in a sequence to weigh the importance of other tokens when generating contextual representations.

**Low-Rank Adaptation (LoRA):** A parameter-efficient fine-tuning technique that injects small trainable low-rank matrices into frozen model layers, enabling domain adaptation without updating the full model.

**Parameter-Efficient Fine-Tuning (PEFT):** A class of methods that adapt large pre-trained models to specific tasks by modifying only a small subset of parameters rather than retraining the entire network.

**LoRA Adapter Fusion:** A strategy that combines multiple task-specific LoRA adapters into a single unified model through weighted merging.

**Layer-wise Merging:** A method of combining model parameters or adapters by interpolating corresponding layers across models using weighted coefficients.

**Output Ensembling:** An inference strategy that aggregates predictions from multiple models, often using voting or similarity-based selection, to improve robustness and reliability.

**Quantization (4-bit Quantization):** A model compression technique that reduces the numerical precision of weights (e.g., from 16-bit or 32-bit to 4-bit) in order to decrease memory usage and inference latency.

**SentencePiece:** A subword tokenization algorithm used to segment text into smaller units for efficient processing by language models.

**Majority Voting:** An ensemble decision method in which the most frequently predicted output among multiple models is selected as the final result.

**BLEU (Bilingual Evaluation Understudy):** A precision-based metric that measures n-gram overlap between generated text and reference text.

**ROUGE-L:** A recall-oriented metric based on the longest common subsequence between generated and reference text, commonly used for evaluating text generation quality.

**BERTScore:** A semantic similarity metric that computes cosine similarity between contextual embeddings of generated and reference texts.

**BioBERT Similarity:** A domain-specific semantic similarity metric derived from embeddings produced by BioBERT, a model pre-trained on biomedical literature.

**Causal Language Modeling (Causal LM):** A generative modeling objective in which the model predicts the next token in a sequence given the preceding tokens.

**Human-in-the-Loop (HITL):** A validation framework in which human experts review, approve, or correct model outputs to enhance reliability and safety.

**Semantic Similarity:** A measure of how closely two pieces of text align in meaning, even if their wording differs.

**Inference Latency:** The time required for a model to generate an output after receiving an input query.

**Data Leakage (Pretraining Overlap):** The unintended exposure of evaluation or benchmark data during model pretraining, which may inflate performance through recall rather than genuine reasoning.
